# Supplementary material for: BRCA2 Regulates Transcription Elongation by RNA Polymerase II to Prevent R-Loop Accumulation
Source: Cell Rep. 2018 Jan 28;22(4):1031–9. doi: 10.1016/j.celrep.2017.12.086 (PMC5846855; doi:10.1016/j.celrep.2017.12.086)
Supplement: Document S1. Figures S1–S3 and Tables S1–S3 [file mmc1.pdf]

Cell Reports, Volume *22*

Supplemental Information

BRCA2 Regulates Transcription Elongation by RNA Polymerase II to Prevent R-Loop Accumulation

Mahmud K.K. Shivji, Xavier Renaudin, Çiğdem H. Williams, and Ashok R. Venkitaraman

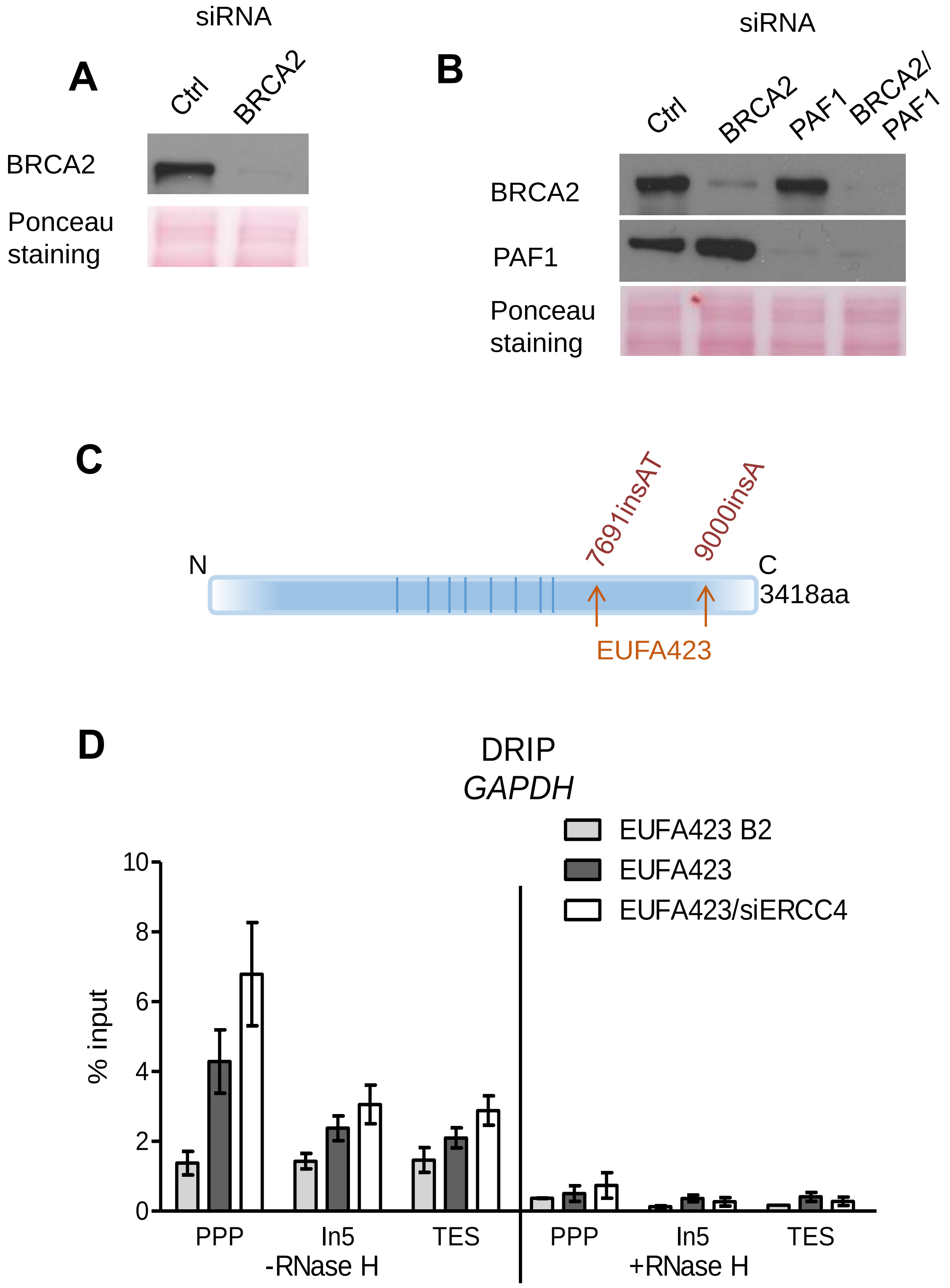

Figure S1. Related to Figures 1, 2 and 6.

(**A**)(**B**) Western blots for BRCA2 or PAF1 in HeLa Kyoto cells treated with the indicated siRNAs. (**C**) Schematic showing the location in the 3418aa human BRCA2 protein of the truncating mutations *BRCA2*^{7691insAT} and *BRCA2*^{9000insA} present in patient-derived EUFA423 cells. EUFA423 B2 control cells have been reconstituted with full-length wild-type BRCA2. (**D**) DRIP analyses with S9.6 antibody of the *GAPDH* gene in EUFA423 B2 and EUFA423 cells, without or with siRNA targeting ERCC4. R-loop digestion by RNAse H enzyme is shown as a control. Plots show the mean±s.e.m from two independent experiments.

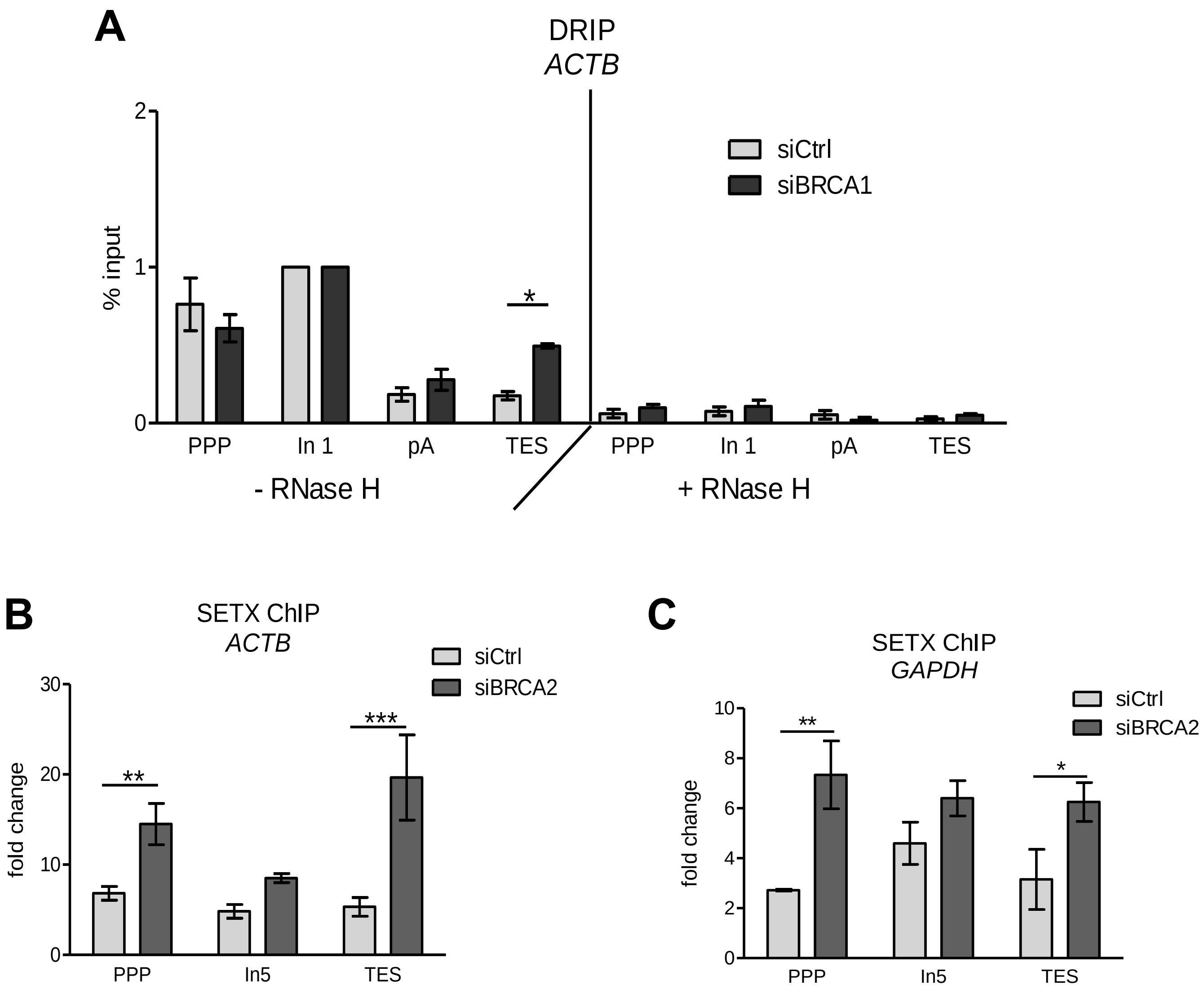

Figure S2. Related to Figure 2.

(**A**) DRIP analysis with S9.6 antibody of ACTB gene in HeLa Kyoto cells transfected with indicated siCtrl or siBRCA1 for 72h. R-loop dissolution by RNAse H enzyme is shown as a control. Error bars indicate the mean±s.e.m from three independent experiments. The 2-way ANOVA test was performed for all pairwise comparisons to determine statistical significance. Statistically significant differences are indicated. Data are normalized to In1. (**B**) ChIP analysis with SETX antibody of *ACTB* gene, and (**C**) *GAPDH* gene in HeLa Kyoto cells transfected with siCtrl or siBRCA2 for 72h. Error bars indicate the mean±s.e.m of three independent experiments. The 2-way ANOVA test was performed for all pairwise comparisons to determine statistical significance. Statistically significant differences are indicated *p<0.05, **p<0.01, ***p<0.001.

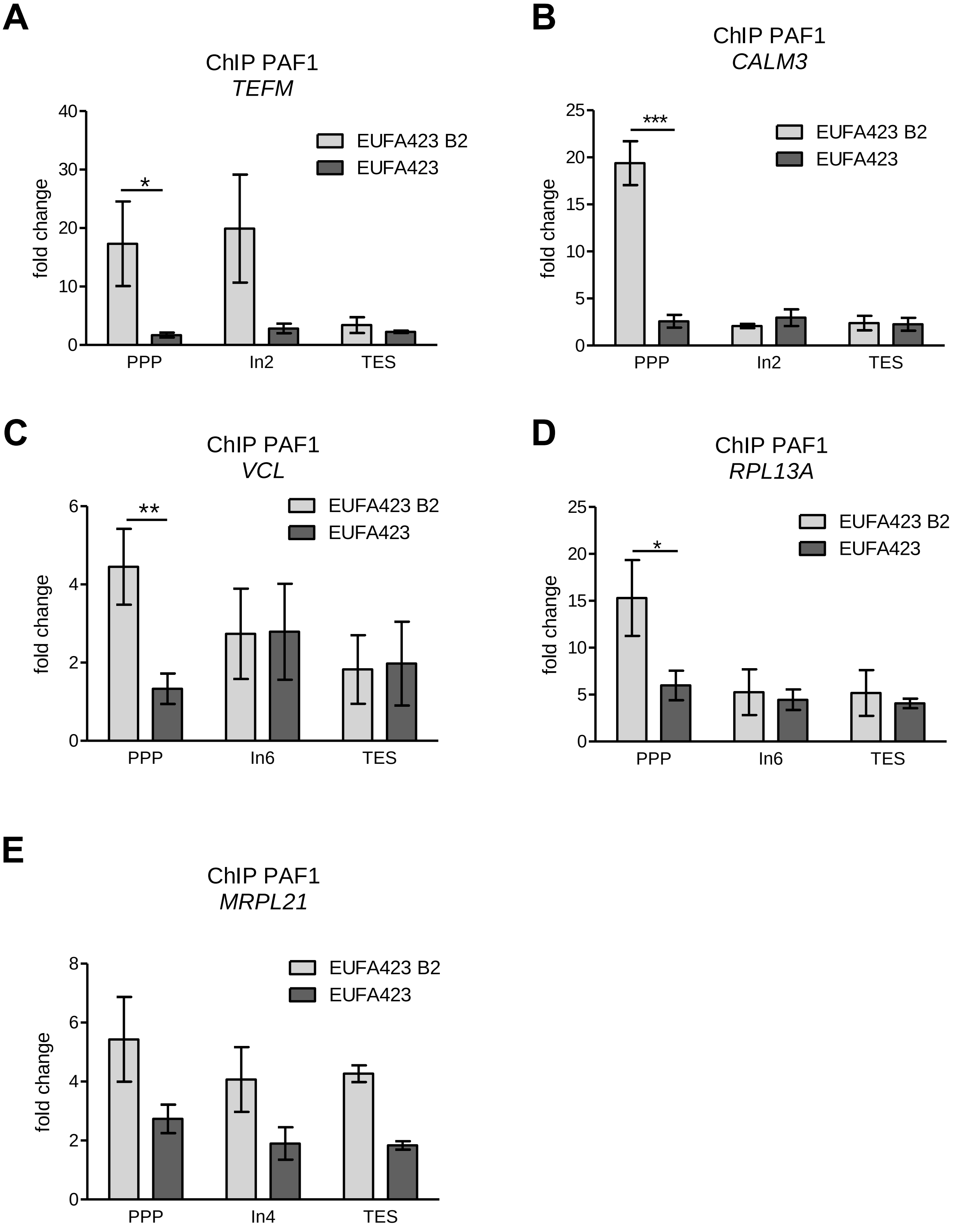

Figure S3. Related to Figure 5.

ChIP analyses of (**A**) *TEFM*, (**B**) *CALM3*, (**C**) *VCL*, (**D**) *RPL13A* and (**E**) *MRPL21* genes with PAF1 antibody in EUFA423 B2 and EUFA423 cells. The 2-way ANOVA test was performed for all pairwise comparisons to determine statistical significance. Statistically significant differences are indicated *$p<0.05$, **$p<0.01$, ***$p<0.001$.

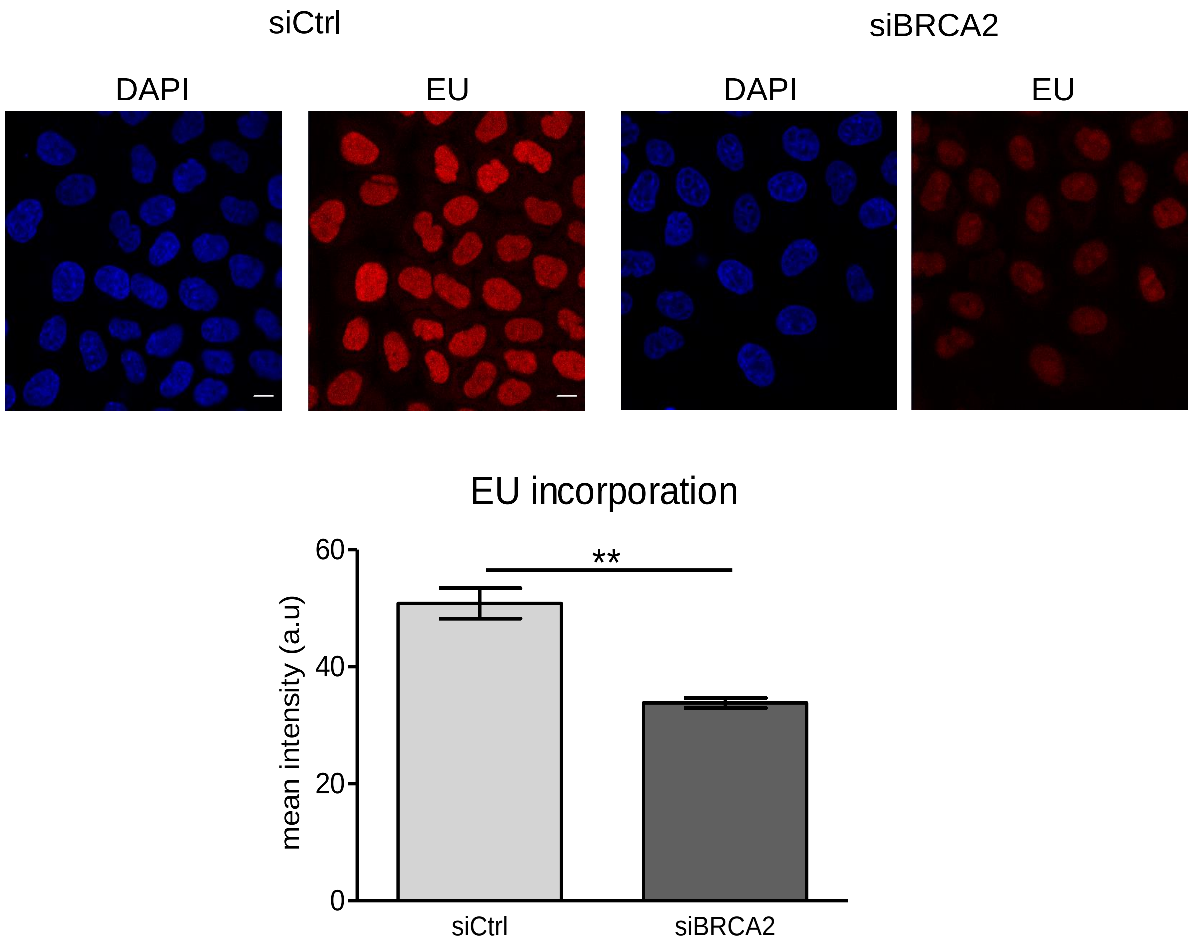

Figure S4. Related to Figure 5.

Immunofluorescence of siCtrl or siBRCA2 transfected HeLa Kyoto cells labelled with the nucleotide analogue 5-Ethynyl Uridine (EU) for 30 minutes to capture nascent RNA synthesis. Scale bars 10µm. The decrease in EU incorporation was quantitated in three independent experiments, and plotted in the bar graph as mean±s.e.m. The two-tailed Student's t-test was performed to determine statistical significance between the two groups. ** indicates $p<0.01$.

Supplemental Table S1. Related to Figures 1-6. Antibodies used in this study.

Antibody name	Clone No:	Cat. No:	Supplier	Stock Conc mg/ml	Application	Host	Clonality
BRCA2	Ab-1	OP95	Merck		WB/IP/ChIP	Mouse	mAb
H2B K120 Ubiquitin	D11	5546S	Cell Signalling		WB/ChIP	Mouse	mAb
H2B		Ab1790	Abcam		WB	Rabbit	pAb
γH2AX (phospho S139)		Ab2893	Abcam		ChIP	Rabbit	pAb
PAF1		Ab137519	Abcam		WB	Rabbit	pAb
PAF1		A300-173A	Bethyl	1ug/uL	ChIP	Rabbit	pAb
RNA polymerase II	MABI0601	MABI0601	MBL Itl	1ug/uL	ChIP	Mouse	mAb
RNA polymerase II **DISCONTINUED**	N20	SC-899	Santa Cruz		WB/IP/ChIP	Rabbit	pAb
RNA polymerase II(Ser5P)	3E8	04-1572	Millipore	1ug/uL	WB/ChIP	Rat	pAb
RNA polymerase II(Ser2P)	3E10	04-1571	Millipore	1ug/uL	WB	Rat	pAb
S9.6			In-house purified	0.84ug/ml	IF (1:100) IP	Mouse	mAb
SETX	QQ7	SC-100319	Santa Cruz		WB/IP/ChIP	Mouse	mAb
SETX		NB100-57542	Novus	1 µg/µl	WB (1:4000)	Rabbit	mAb

Supplemental Table S2. Related to Figures 1-6. Primers used in this study.

	Forward	Reverse
ACTB_PPP	GAGGGGAGAGGGGGTAAA	AGCCATAAAAGGCAACTTTCG
ACTB_In1	CGGGGTCTTTGTCTGAGC	CAGTTAGCGCCCAAAGGAC
ACTB_In5	GGAGCTGTCACATCCAGGGTC	TGCTGATCCACATCTGCTGG
ACTB_pA	TTACCCAGAGTGCAGGTGTG	CCCCAATAAGCAGGAACAGA
ACTB_TES	GGGACTATTTGGGGGTGTCT	TCCCATAGGTGAAGGCAAAG
GAPDH_PPP	CTCCTGTTCGACAGTCAGC	TTCAGGCCGTCCCTAGC
GAPDH_In5	ATAGGCGAGATCCCTCCAA	TGAAGACGCCAGTGGAC
GAPDH_TES	CCCTGTGCTCAACCAGT	CTCACCTTGACACAAGCC
TEFM_PPP	CTTGGAGATGAGCGGGTCTG	GACAGACGGGAAATCACCCC
TEFM_In2	TGGCCAATGTGGTGAAAGCC	GGGACTACAGGCCCACGCC
TEFM_TES	ACCACATAGACTTTATGACAGAGAA	TCAATCCATGCTTGTGAAGCAAA
CALM3_PPP	TGCGGGCAGTGAGTGTGGAGG	ACGGGGATCAAGGTTCCTCCGG
CALM3_In1	GTTCGGGCCCCTATTGCGCAC	AAAAGCTGGCTCATTCGAGGCACC
CALM3_TES	GCGATGCCCGTTCTCTTGATC	CGCAGGGGAGTGTTGAAGAGAGA
MRPL21_PPP	CGTTACGCACGCGGTTC	GACCGTCAGGGAAGATGCTG
MRPL21_In4	CGGCATTGAGAATGGTTGCC	GCAATGATTGAGGCTCTCCT
MRPL21_TES	ACCAGGTTTCTGTGTTCTGGT	GGCCTGGTGCTTACAGACAT
RPL13A_PPP	ATGGCGGAGGTGCAGGTATG	AGAGAGGGTGCGACCCCATT
RPL13A_In6	AGATTTCAGGCCTGCTGAGG	CCGCAGACCATCGTGAGATA
RPL13A_TES	TGGCGTCTTTGCACTGTGTC	CTACCCTCTTCAAGCTCCTCAC
VCL_PPP	GTGAGGCTGGTTACGCCGAG	CGGGAACCGGCGAAGAGA
VCL_In6	CTCTGGTATCTGAATCTGCTTTCT	AGCAGTATTTGCAATGTTTGGTTT
VCL_TES	AGAACTTTTAGGTCAGGTTTCTCCT	GTTTCCAGATCTTGAGGATTAGTTT

Supplemental Table S3. Related to Figures 1-3, 5-6, S2-S4. Statistical analysis.

**Figure 1**			P value	summary							
**A**	t-test	two-tailed	0.0061	**							
**B**	t-test	two-tailed	0.0087	**							
**C left**						**C right**					
EUFA423 B2 vs EUFA423	Row Factor	Difference	t	P value	Summary	siCtrl vs siBRCA2	Row Factor	Difference	t	P value	Summary
2-way ANOVA	PPP	0.62	3.918	P<0.01	**	2-way ANOVA	PPP	11.67	3.198	P < 0.05	*
2-way ANOVA	In1	0.18	1.137	P > 0.05	ns	2-way ANOVA	In1	-1	0.2741	P > 0.05	ns
2-way ANOVA	pA	-0.125	0.645	P > 0.05	ns	2-way ANOVA	pA	1.667	0.4569	P > 0.05	ns
2-way ANOVA	TES	0.06	0.3792	P > 0.05	ns		TES	2.667	0.7311	P > 0.05	ns
**D left**						**D right**					
EUFA423 B2 vs EUFA423	Row Factor	Difference	t	P value	Summary	siCtrl vs siBRCA2	Row Factor	Difference	t	P value	Summary
2-way ANOVA	PPP	2.3	2.802	P < 0.05	*	2-way ANOVA	PPP	5.767	5.449	P<0.001	***
2-way ANOVA	In5	0.4833	0.5889	P > 0.05	ns	2-way ANOVA	In5	0.03333	0.0315	P > 0.05	ns
2-way ANOVA	TES	0.33	0.4021	P > 0.05	ns	2-way ANOVA	TES	0.6	0.5669	P > 0.05	ns
**E left**						**E right**					
EUFA423 B2 vs EUFA423	Row Factor	Difference	t	P value	Summary	siCtrl vs siBRCA2	Row Factor	Difference	t	P value	Summary
2-way ANOVA	PPP	1.06	0.8644	P > 0.05	*	2-way ANOVA	PPP	30.71	3.878	P<0.01	**
2-way ANOVA	In2	0.16	0.1305	P > 0.05	ns	2-way ANOVA	In2	9.39	1.186	P > 0.05	ns
2-way ANOVA	TES	3.333	2.718	P > 0.05	ns	2-way ANOVA	TES	2.733	0.3451	P > 0.05	ns

**Figure 2**											
**A**						**B**					
EUFA423 B2 vs EUFA423	Row Factor	Difference	t	P value	Summary	EUFA423 B2 vs EUFA423					
2-way ANOVA	PPP	0.835	4.796	P<0.01	**	2-way ANOVA	Row Factor	Difference	t	P value	Summary
2-way ANOVA	In4	-0.05	0.2872	P > 0.05	ns	2-way ANOVA	PPP	7.668	3.998	P<0.01	**
2-way ANOVA	TES	-0.375	2.154	P > 0.05	ns	2-way ANOVA	In2	-0.335	0.1747	P > 0.05	ns
**C**						**D**					
EUFA423 B2 vs EUFA423	Row Factor	Difference	t	P value	Summary	EUFA423 B2 vs EUFA423	Row Factor	Difference	t	P value	Summary
2-way ANOVA	PPP	0.51	3.964	P<0.01	**	2-way ANOVA	PPP	4.765	5.337	P<0.01	**
2-way ANOVA	In6	-0.05667	0.4404	P > 0.05	ns	2-way ANOVA	In6	1.81	2.027	P > 0.05	ns
2-way ANOVA	TES	0.09667	0.7513	P > 0.05	ns	2-way ANOVA	TES	0.39	0.4368	P > 0.05	ns
**E**						**E**					
EUFA423 B2 vs EUFA423	Row Factor	Difference	t	P value	Summary	EUFA423 vs EUFA423/ERRC4	Row Factor	Difference	t	P value	Summary
2-way ANOVA	PPP	1.368	4.925	P<0.01	**	2-way ANOVA	PPP	-2.123	7.335	P<0.001	***
2-way ANOVA	In5	0.7926	2.853	P < 0.05	*	2-way ANOVA	In5	-1.493	5.158	P<0.001	***
2-way ANOVA	TES	0.7592	2.733	P > 0.05	ns	2-way ANOVA	TES	-1.228	4.241	P<0.01	**

**Figure 3**											
**A left**						**A right**					
EUFA423 B2 vs EUFA423	Row Factor	difference	t	P value	summary	EUFA423 B2 vs EUFA423	Row Factor	Difference	t	P value	Summary
2-way ANOVA	PPP	184.3	3.396	P < 0.05	*	2-way ANOVA	PPP	37.67	3.067	P < 0.05	*
2-way ANOVA	In5	2	0.0369	P > 0.05	ns	2-way ANOVA	In5	2.5	0.1662	P > 0.05	ns
2-way ANOVA	TES	62.33	1.148	P > 0.05	ns	2-way ANOVA	TES	0.3333	0.0272	P > 0.05	ns
**B left**						**B right**					
EUFA423 B2 vs EUFA423	Row Factor	Difference	t	P value	Summary	EUFA423 B2 vs EUFA423	Row Factor	Difference	t	P value	Summary
2-way ANOVA	PPP	-0.1767	1.791	P > 0.05	ns	2-way ANOVA	PPP	-0.028	0.4378	P > 0.05	ns
2-way ANOVA	In5	-0.1333	1.352	P > 0.05	ns	2-way ANOVA	In5	-0.099	1.548	P > 0.05	ns
2-way ANOVA	TES	0.04333	0.4394	P > 0.05	ns	2-way ANOVA	TES	-0.09	1.407	P > 0.05	ns

**Figure 5**											
**A left**						**A right**					
siCtrl vs siBRCA2	Row Factor	difference	t	p-value	summary	siCtrl vs siBRCA2	Row Factor	difference	t	p-value	summary
2-way ANOVA	PPP	-31.06	4.395	P<0.01	**	2-way ANOVA	PPP	-3.75	1.594	P > 0.05	ns
2-way ANOVA	In5	-11.46	1.622	P > 0.05	ns	2-way ANOVA	In5	-14.5	6.163	P<0.01	**
2-way ANOVA	TES	-14.94	2.114	P > 0.05	ns	2-way ANOVA	TES	-10.15	4.314	P < 0.05	*
**B left**						**B right**					
EUFA423 B2 vs EUFA423	Row Factor	difference	t	p-value	summary	EUFA423 B2 vs EUFA423	Row Factor	difference	t	p-value	summary
2-way ANOVA	PPP	11.67	3.198	P < 0.05	*	2-way ANOVA	PPP	-26.37	4.626	P<0.01	**
2-way ANOVA	In5	-14.55	2.143	P > 0.05	ns	2-way ANOVA	In5	-27.3	4.79	P<0.01	**
2-way ANOVA	TES	-13.29	1.958	P > 0.05	ns	2-way ANOVA	TES	-40.43	7.095	P<0.001	***
**C**						**D**					
siCtrl vs siBRCA2	Row Factor	difference	t	p-value	summary	EUFA423 B2 vs EUFA423	Row Factor	difference	t	p-value	summary
2-way ANOVA	PPP	0	0	P > 0.05	ns	2-way ANOVA	PPP	0	0	P > 0.05	ns
2-way ANOVA	In5	-0.5025	6.185	P<0.001	***	2-way ANOVA	In5	-0.7236	3.049	P < 0.05	*
2-way ANOVA	TES	-0.4417	5.436	P<0.001	***	2-way ANOVA	TES	-0.7155	3.015	P < 0.05	*

**Figure 6**					
**A**					
siCtrl vs siBRCA2	Row Factor	Difference	t	P value	Summary
2-way ANOVA	PPP	6.888	1.707	P < 0.05	*
2-way ANOVA	In5	1.533	0.4103	P > 0.05	ns
2-way ANOVA	TES	1.095	0.2931	P > 0.05	ns
siCtrl vs siPAF1	Row Factor	Difference	t	P value	Summary
2-way ANOVA	PPP	12.06	3.229	P<0.01	**
2-way ANOVA	In5	5.233	1.401	P > 0.05	ns
2-way ANOVA	TES	4.67	1.25	P > 0.05	ns
siCtrl vs siBRCA2/siPAF1	Row Factor	Difference	t	P value	Summary
2-way ANOVA	PPP	11.09	2.748	P < 0.05	*
2-way ANOVA	In5	2.333	0.5781	P > 0.05	ns
2-way ANOVA	TES	1.812	0.449	P > 0.05	ns
**C**					
EUFA423 B2 vs EUFA423	Row Factor	Difference	t	P value	Summary
2-way ANOVA	PPP	3.015	3.78	P < 0.05	*
2-way ANOVA	In5	-0.15	0.1881	P > 0.05	ns
2-way ANOVA	TES	-0.4	0.5015	P > 0.05	ns
EUFA423 B2 vs EUFA423 + PAF1	Row Factor	Difference	t	P value	Summary
2-way ANOVA	PPP	-0.09	0.1128	P > 0.05	ns
2-way ANOVA	In5	-0.885	1.11	P > 0.05	ns
2-way ANOVA	TES	-1.38	1.73	P > 0.05	ns

Supplemental Figure 2

A

siCtrl vs siBRCA1	Row Factor	Difference	t	P value	Summary
2-way ANOVA	PPP	-0.1537	1.474	P > 0.05	ns
2-way ANOVA	In 1	0	0	P > 0.05	ns
2-way ANOVA	pA	0.09445	0.9058	P > 0.05	ns
2-way ANOVA	TES	0.319	3.06	P < 0.05	*

B

siCtrl vs siBRCA2	Row Factor	Difference	t	P value	Summary
2-way ANOVA	PPP	4.615	4.999	P<0.01	**
2-way ANOVA	In5	1.8	1.95	P > 0.05	ns
2-way ANOVA	TES	3.1	3.358	P < 0.05	***

C

siCtrl vs siBRCA2	Row Factor	Difference	t	P value	Summary
2-way ANOVA	PPP	7.667	4.193	P<0.01	**
2-way ANOVA	In5	3.667	2.006	P > 0.05	ns
2-way ANOVA	TES	14.33	7.84	P < 0.05	*

Supplemental Figure 3

A

EUFA423 B2 vs EUFA423	Row Factor	Difference	t	P value	Summary
2-way ANOVA	PPP	-15.64	2.84	P < 0.05	*
2-way ANOVA	In2	-17.09	2.776	P > 0.05	ns
2-way ANOVA	TES	-1.16	0.2107	P > 0.05	ns

B

EUFA423 B2 vs EUFA423	Row Factor	Difference	t	P value	Summary
2-way ANOVA	PPP	-16.81	10.4	P<0.001	***
2-way ANOVA	In2	0.8933	0.5528	P > 0.05	ns
2-way ANOVA	TES	-0.1267	0.0784	P > 0.05	ns

C

EUFA423 B2 vs EUFA423	Row Factor	Difference	t	P value	Summary
2-way ANOVA	PPP	-3.123	3.874	P<0.01	**
2-way ANOVA	In6	0.05333	0.0662	P > 0.05	ns
2-way ANOVA	TES	0.15	0.186	P > 0.05	ns

D

EUFA423 B2 vs EUFA423	Row Factor	Difference	t	P value	Summary
2-way ANOVA	PPP	-9.317	2.844	P < 0.05	*
2-way ANOVA	In6	-0.81	0.2473	P > 0.05	ns
2-way ANOVA	TES	-1.113	0.3399	P > 0.05	ns

Supplemental Figure 4

Unpaired t test	
P value	0.0035
P value summary	**
