# Supplementary material for: BRCA2 Regulates Transcription Elongation by RNA Polymerase II to Prevent R-Loop Accumulation
Source: Cell Rep. 2018 Jan 28;22(4):1031–9. doi: 10.1016/j.celrep.2017.12.086 (PMC5846855; doi:10.1016/j.celrep.2017.12.086)
Supplement: Document S2. Article plus Supplemental Information [file mmc2.pdf]

Cell Reports

Article

BRCA2 Regulates Transcription Elongation by RNA Polymerase II to Prevent R-Loop Accumulation

Graphical Abstract

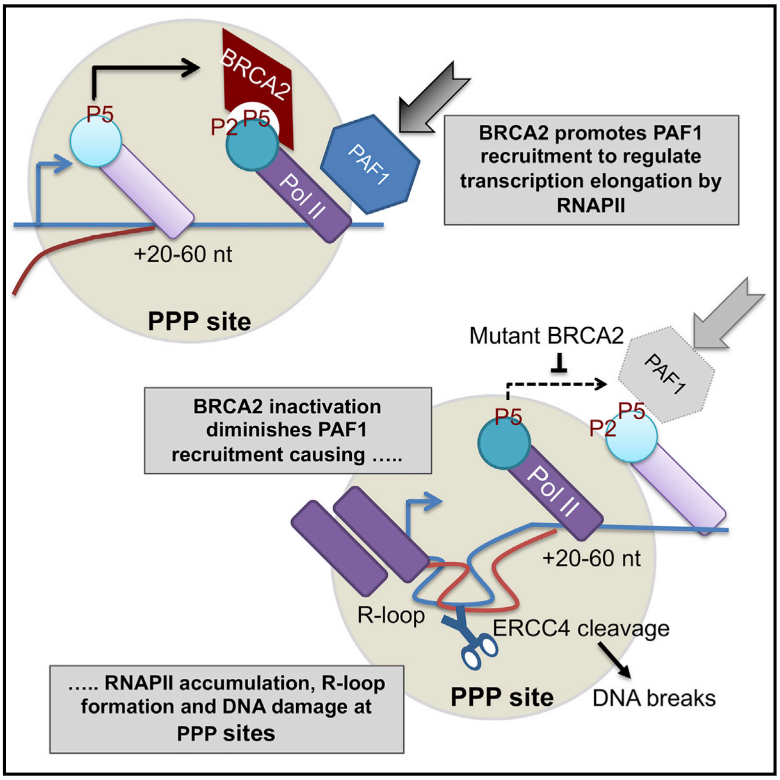

Highlights

- BRCA2 binds RNAPII to regulate promoter-proximal pausing via PAF1 recruitment
- R-loops accrue at PPP sites after BRCA2 inactivation, causing DNA breakage by ERCC4
- PAF1 depletion phenocopies, while PAF1 overexpression ameliorates, BRCA2 deficiency
- Thus, BRCA2 inactivation induces widespread DNA damage via defective RNAPII control

Authors

Mahmud K.K. Shivji, Xavier Renaudin, Çiğdem H. Williams, Ashok R. Venkitaraman

Correspondence

arv22@mrc-cu.cam.ac.uk

In Brief

Shivji et al. find that the BRCA2 tumor suppressor controls transcription elongation by RNA polymerase II. Cancer-associated BRCA2 mutations diminish PAF1 recruitment to the polymerase, provoking unscheduled R-loop accumulation at promoter-proximal pause sites and triggering DNA breakage. This mechanism may underlie genome instability and carcinogenesis after BRCA2 inactivation.

Shivji et al., 2018, Cell Reports *22*, 1031–1039
January 23, 2018 © 2017 The Authors.
https://doi.org/10.1016/j.celrep.2017.12.086

OPEN ACCESS
CellPress

BRCA2 Regulates Transcription Elongation by RNA Polymerase II to Prevent R-Loop Accumulation

Mahmud K.K. Shivji,[1,2] Xavier Renaudin,[1,2] Çiğdem H. Williams,[1] and Ashok R. Venkitaraman[1,3,*]
[1]Medical Research Council Cancer Unit, University of Cambridge, Hills Road, Cambridge CB2 0XZ, UK
[2]These authors contributed equally
[3]Lead Contact
*Correspondence: arv22@mrc-cu.cam.ac.uk
https://doi.org/10.1016/j.celrep.2017.12.086

SUMMARY

The controlled release of RNA polymerase II (RNAPII) from promoter-proximal pausing (PPP) sites is critical for transcription elongation in metazoans. We show that the human tumor suppressor BRCA2 interacts with RNAPII to regulate PPP release, thereby preventing unscheduled RNA-DNA hybrids (R-loops) implicated in genomic instability and carcinogenesis. BRCA2 inactivation by depletion or cancer-causing mutations instigates RNAPII accumulation and R-loop accrual at PPP sites in actively transcribed genes, accompanied by γH2AX formation marking DNA breakage, which is reduced by ERCC4 endonuclease depletion. BRCA2 inactivation decreases RNAPII-associated factor 1 (PAF1) recruitment (which normally promotes RNAPII release) and diminishes H2B Lys120 ubiquitination, impeding nascent RNA synthesis. PAF1 depletion phenocopies, while its overexpression ameliorates, R-loop accumulation after BRCA2 inactivation. Thus, an unrecognized role for BRCA2 in the transition from promoter-proximal pausing to productive elongation via augmented PAF1 recruitment to RNAPII is subverted by disease-causing mutations, provoking R-loop-mediated DNA breakage in *BRCA2*-deficient cells.

INTRODUCTION

Inherited mutations inactivating the *BRCA2* tumor suppressor gene predispose to cancers of the breast, ovary, pancreas, prostate, and other tissues (Breast Cancer Linkage Consortium, 1999). Human BRCA2 protein serves as a custodian of chromosome integrity via the nucleation of multi-protein complexes essential for homologous DNA recombination, replication fork protection, and cell-cycle control (reviewed in Venkitaraman, 2014). The chromosomal instability characteristic of *BRCA2*-deficient cells has been attributed to loss of these functions, but recent findings show that *BRCA2* deficiency also causes the unscheduled accumulation of RNA-DNA hybrids (R-loops) (Bhatia et al., 2014) and link R-loop accrual to chromosomal structural aberrations in *BRCA2*-deficient cells (Tan et al., 2017).

The mechanism by which BRCA2 deficiency causes R-loop accumulation remains unclear (Bhatia et al., 2014). R-loops are normal intermediates during processes like transcription, replication, or DNA repair, but they also occur when RNA polymerase II (RNAPII) stalls at nucleotide lesions that arrest the transcription of DNA templates (Aguilera and García-Muse, 2012; Hamperl and Cimprich, 2014; Sollier and Cimprich, 2015). In addition, RNAPII pausing occurs physiologically during an early step in transcription elongation to efficiently manage gene expression patterns that suit cellular requirements (Jonkers and Lis, 2015). Most RNAPII-transcribed genes in metazoan cells contain a promoter-proximal pause (PPP) site located ∼20-50 nucleotides downstream of the position where transcription begins (Krumm et al., 1995; Rasmussen and Lis, 1993; Rougvie and Lis, 1988). RNAPII enriched for phosphorylation on Ser5 in its C-terminal domain (CTD) pauses at these sites after the synthesis of a short nascent RNA tract (Boehm et al., 2003). RNAPII release from PPP sites is accompanied by the phosphorylation of Ser2 in its CTD by the kinase cyclin T-CDK9 (Cho et al., 2001; Marshall et al., 1996), empowering the recruitment of a protein complex including RNAPII-associated factor 1 (PAF1) (Chen et al., 2015; Yu et al., 2015). In turn, PAF1 recruitment to RNAPII triggers events leading to the ubiquitination of histone H2B on Lys120, a modification that is proposed to open chromatin downstream of the transcription complex to facilitate transcription elongation (Van Oss et al., 2016; Wu et al., 2014; Xiao et al., 2005). Mounting evidence suggests that RNAPII release from PPP sites is tightly regulated to control mammalian gene expression (reviewed in Adelman and Lis, 2012).

Inactivation of BRCA1, a tumor suppressor protein that is functionally and structurally distinct from BRCA2 but cooperates with it during cellular responses to DNA damage (reviewed in Venkitaraman, 2014), is also accompanied by unscheduled R-loop accumulation (Hatchi et al., 2015; Zhang et al., 2017). A small pool of intracellular BRCA1 interacts with RNAPII (Schlegel et al., 2000; Scully et al., 1997). Conflicting reports variously attribute R-loop formation in BRCA1-deficient cells either to transcription termination sites at the 3′ ends of genes (Hatchi et al., 2015) via the interaction of BRCA1 with the R-loop resolvase senataxin (SETX) (Skourti-Stathaki et al., 2011) or, alternatively, to 5′ PPP sites (Zhang et al., 2017) only in certain epithelial cell types through mechanisms that remain unclear. Moreover, whether R-loop accrual following BRCA2 inactivation arises from similar anomalies is not known.

Here, we report an unanticipated role for BRCA2 in the control of transcription elongation at PPP sites through the enhanced

This is an open access article under the CC BY license (http://creativecommons.org/licenses/by/4.0/).

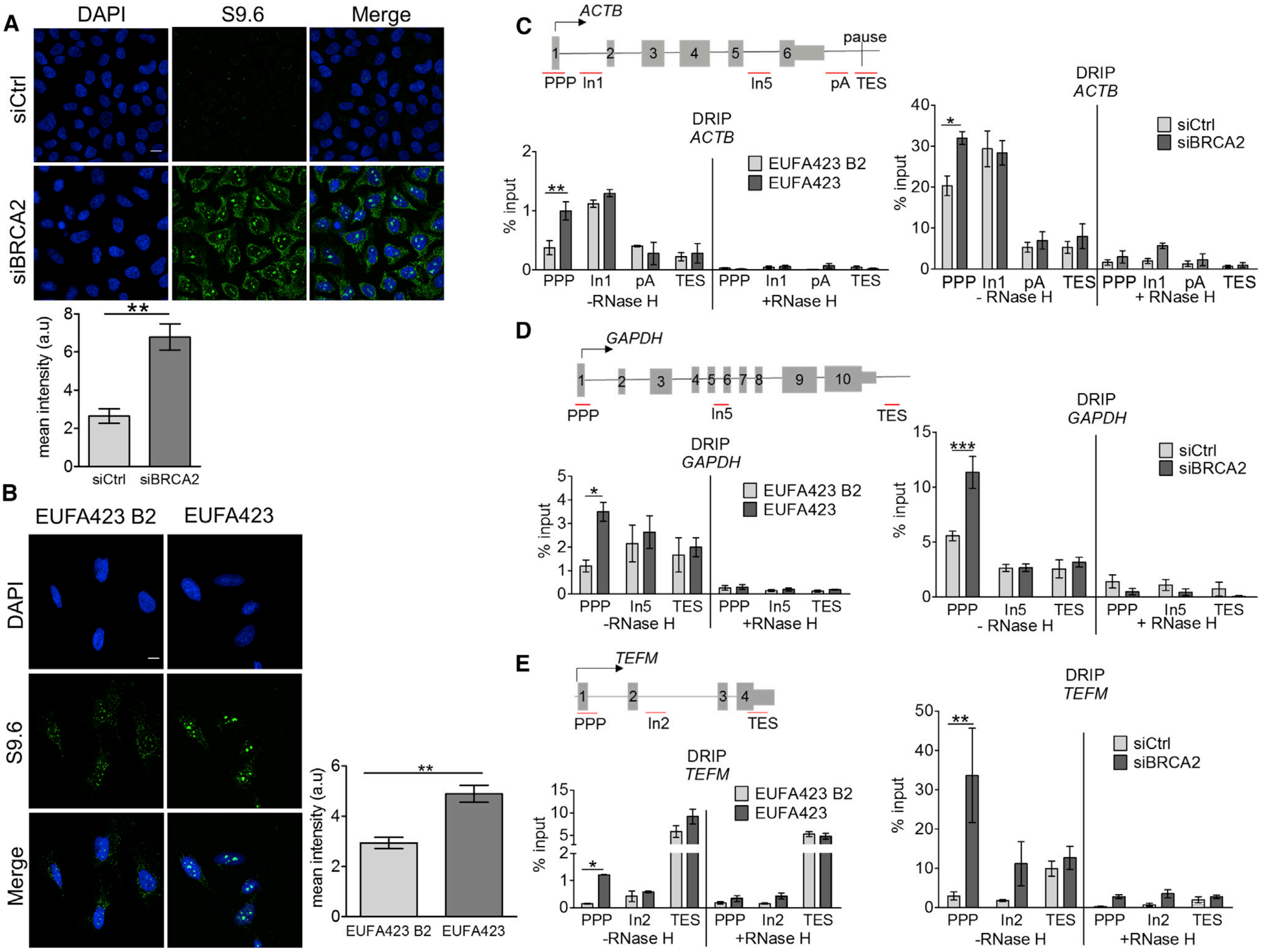

Figure 1. BRCA2 Deficiency Leads to R-Loop Formation Accompanied by RNAPII Accumulation at the PPP Sites of Classical Paused Genes

(A and B) Immunofluorescence detection of R-loops with S9.6 antibody in HeLa Kyoto cells transfected with either siCtrl (control) or siBRCA2 (A) and BRCA2-deficient EUFA423 cells (see Figure S1C) and EUFA423 B2 controls complemented with wild-type BRCA2 (B). HeLa cell transfections were carried out for 72 hr before analysis. Plots show the mean ± SEM from three independent experiments. The two-tailed Student's t test was performed to determine statistical significance between the two groups (**p < 0.01). Scale bars, 10 μm.

(C) Schematic diagram of the *ACTB* gene showing primer positions (PPP, In1 or In5, pA, and TES) and exon numbers. DRIP analyses with S9.6 antibody in EUFA423 B2 and EUFA423 (left) and in HeLa Kyoto cells transfected with the indicated siRNA for 72 hr (right) are shown. R-loop digestion by RNase H is shown as a control. Plots depict the mean ± SEM from three independent experiments. The 2-way ANOVA test was performed for all pairs to determine statistical significance. Statistically significant differences are indicated (**p < 0.01, left; *p < 0.05, right).

(D) Schematic diagram of the *GAPDH* gene, showing primer positions (PPP, In5, and TES) and exon numbers. DRIP analyses were performed and depicted as described in the preceding panels. Statistically significant differences are indicated (*p < 0.05, left; ***p < 0.001, right).

(E) Schematic diagram of the *TEFM* gene, showing primer positions (PPP, In2 and TES) and exon numbers. DRIP analyses were performed and depicted as described in the preceding panels. Statistically significant differences are indicated (*p < 0.05, left; **p < 0.01, right).

recruitment of PAF1 to RNAPII. BRCA2 inactivation by cancer-associated mutations or RNAi-mediated depletion subverts this mechanism, impeding transcription elongation at the PPP sites of actively transcribed genes, accompanied by R-loop accumulation and DNA breakage mediated by the ERCC4 nuclease. By contrast, BRCA1 or SETX depletion causes R-loop accumulation at 3′ transcription termination sites, distinguishing the mechanism mediated by BRCA2. Thus, our work identifies an unrecognized mechanism by which BRCA2 controls transcription elongation and suggests that abnormalities in this process instigate unscheduled R-loop formation and ensuing genomic instability.

RESULTS

BRCA2 Inactivation Triggers R-Loop and DNA Damage Accumulation at PPP Sites

Nuclear R-loop accumulation detected by the S9.6 antibody specific to R-loop structures (Bhatia et al., 2014; Ginno et al., 2012) increases following BRCA2 inactivation by small interfering RNA (siRNA)-mediated depletion in HeLa Kyoto cells (Figures 1A and S1A) or by bi-allelic cancer-associated truncating mutations (*BRCA2*$^{7691insAT / 9000insA}$) in the patient-derived EUFA423 fibroblast cell line (Howlett et al., 2002) (Figures 1B and

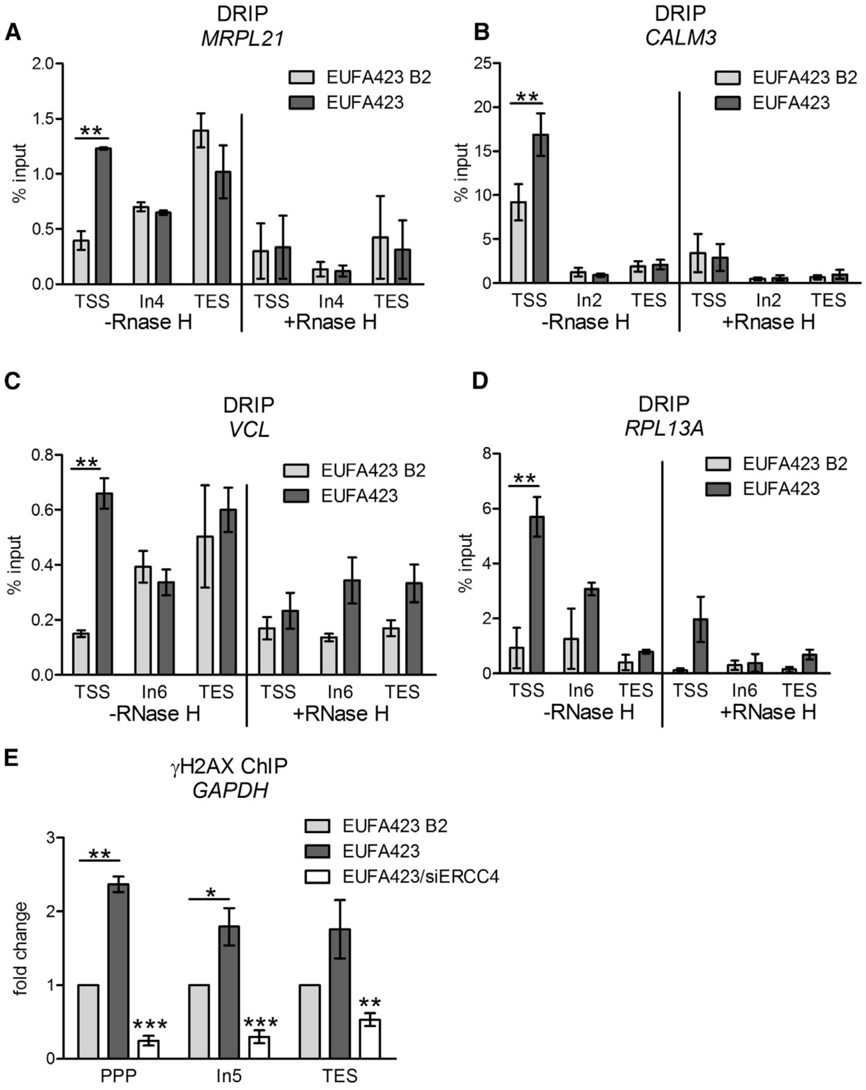

Figure 2. R-Loops Accumulate at the PPP Sites of Multiple Genes in BRCA2-Deficient Cells

(A–D) DRIP analysis of *MRPL21* (A), *CALM3* (B), *VCL* (C), and *RPL13A* (D) genes in EUFA423 B2 and EUFA423 cells. R-loop dissolution by RNase H enzyme is shown as a control. Error bars indicate the mean ± SEM from three independent experiments. The 2-way ANOVA test was performed for all pairwise comparisons to determine statistical significance. Statistically significant differences are indicated (**p < 0.01).

(E) ChIP analysis with γH2AX antibody of the *GAPDH* gene in EUFA423 B2 and EUFA423 cells, without or with siRNA targeting ERCC4. Fold change relative to EUFA423 B2 is plotted. Error bars indicate the mean ± SEM from three independent experiments. The 2-way ANOVA test was performed for all pairwise comparisons to determine statistical significance. For EUFA423/ERCC4, significance was determined by comparison with EUFA423. Statistically significant differences are indicated (*p < 0.05, **p < 0.01, and ***p < 0.001).

S1C). By contrast, EUFA423 cells stably complemented with full-length FLAG-tagged BRCA2 (EUFA423 B2) (Hattori et al., 2011) show no such abnormality (Figure 1B).

We used S9.6 antibody in DNA-RNA immunoprecipitation (DRIP) analyses (Ginno et al., 2012) to survey R-loop formation in genomic DNA across the transcription units of seven actively transcribed genes: *ACTB*, *GAPDH, TEFM*, *MRPL21*, *CALM3*, *RPL13A*, and *VCL* (Hatchi et al., 2015; Sanz et al., 2016; Zhao et al., 2016). We analyzed R-loop formation at the 5′ PPP sites of these genes within 200 nt of their transcription start site (TSS) sequences, as well as at intronic (In) sequences in the gene body or the 3′ sequences associated with transcription-end sites (TESs). In HeLa Kyoto cells depleted of BRCA2, there is a statistically significant increase in R-loop formation at the PPP sites relative to other regions of *ACTB, GAPDH* and *TEFM* (Figure 1C-E), when compared to cells treated with irrelevant control siRNAs. R-loop formation at the PPP sites of these genes is also increased in BRCA2-deficient EUFA423 cells, in comparison to EUFA423 B2 isogenic controls reconstituted with BRCA2 (Figures 1C–1E). Similar anomalies occur at the PPP sites of *MRPL21, CALM3, VCL* and *RPL13A* genes in EUFA423 when compared to EUFA423 B2 cells (Figures 2A–2D). R-loop detection in these experiments is uniformly suppressed by pretreatment with RNase H (Figures 1C–1E and 2A–2D), an enzyme that digests R-loops (Ginno et al., 2012).

Interestingly, R-loop accumulation in the *GAPDH* transcription unit is accompanied by γH2AX formation, a marker for DNA damage (Figure 2E), most markedly at the PPP site but also at the adjacent intronic region. Depletion of the endonuclease ERCC4 (Sijbers et al., 1996), which has been implicated in the cleavage of R-loops to DNA breaks (Sollier et al., 2014), reduces γH2AX formation (Figure 2E) without significantly altering R-loop accumulation (Figure S1D). These findings suggest a mechanism wherein R-loop processing by ERCC4 contributes to genomic instability in BRCA2-deficient cells.

By contrast, depletion of BRCA1 enhances R-loops in a significant manner at sequences associated with transcription termination (TES) in *ACTB* (Figure S2A), consistent with a previous report (Hatchi et al., 2015), but has little effect on R-loop formation at PPP sites, in contrast to another study (Zhang et al., 2017). These observations are distinct from the defect that we describe for BRCA2 deficiency. Moreover, we find that BRCA2 depletion increases the accumulation of SETX (a helicase implicated in R-loop resolution during transcription termination; Skourti-Stathaki et al., 2011) at PPP sites in *ACTB* and *GAPDH*, as well as in their termination sequences, as detected by chromatin immunoprecipitation (ChIP) (Figures S2B and S2C). This pattern contrasts with the decreased

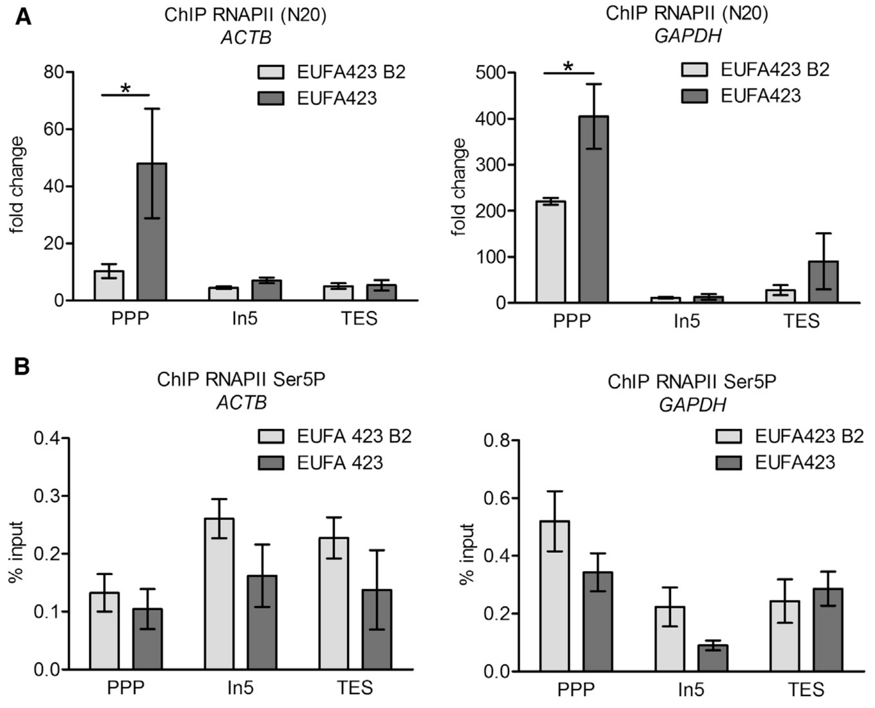

Figure 3. RNAPII Accumulates at the PPP Sites of Actively Transcribed Genes

(A) ChIP analyses with RNAPII antibody (N20) of the *ACTB* and *GAPDH* genes in EUFA423 B2 and EUFA423 cells. Fold change relative to IgG isotype control is plotted. Plots show the mean ± SEM from three independent experiments. The 2-way ANOVA test was performed to determine statistical significance (*p < 0.05).

(B) ChIP analyses of RNAPII Ser5P in the *ACTB* and *GAPDH* genes in EUFA423 B2 and EUFA423 cells. Data were normalized to input (2%). Plots show the mean ± SEM from three independent experiments; the 2-way ANOVA test was performed as above.

recruitment of SETX to transcription termination sequences observed after BRCA1 depletion (Hatchi et al., 2015), again suggesting that R-loop formation after BRCA2 depletion occurs via a distinct mechanism.

BRCA2 Inactivation Causes RNAPII Accumulation at PPP Sites

We next examined the effect of BRCA2 inactivation on the distribution of RNAPII across the *ACTB* and *GAPDH* transcription units using an antibody (N20) (Cheng and Sharp, 2003) capable of detecting both the non-phosphorylated and CTD Ser2/Ser5 phosphorylated forms of the enzyme. RNAPII was significantly enriched at PPP sites (as detected by ChIP) in EUFA423 cells carrying bi-allelic *BRCA2* mutations, but not in control EUFA423 B2 cells reconstituted with full-length BRCA2 (Figure 3A). ChIP using an antibody that selectively binds to the Ser5-phosphorylated form of RNAPII (Chapman et al., 2007) reveals a more even distribution across both genes (Figure 3B), although levels are diminished in *BRCA2*-deficient EUFA423 cells when compared with EUFA423 B2 controls. Collectively, these findings suggest that the release of RNAPII from PPP sites in *ACTB* and *GAPDH* during transcription elongation is delayed, but not stopped.

BRCA2 Interacts with RNAPII

The unexpected effect of BRCA2 inactivation on RNAPII distribution prompted us to test whether these proteins interact. Indeed, RNAPII immunoprecipitated from HeLa Kyoto cell extracts using the N20 antibody interacts with wild-type BRCA2 (Figure 4A). EUFA423 cells carry the bi-allelic $BRCA2^{7691insAT / 9000insA}$ truncation mutations, which encode truncated proteins with predicted molecular weights of 278 kDa ($BRCA2^{7691insAT}$) and 358 kDa ($BRCA2^{9000insA}$), while control EUFA423 B2 cells express, in addition, full-length FLAG-tagged BRCA2 protein of 450 kDa. In EUFA423 B2 cell extracts (Figure 4B, lanes 1–3), RNAPII immunoprecipitated with the N20 antibody interacts with full-length and the $BRCA2^{9000insA}$ truncated mutant proteins, but as expected in EUFA423 cell extracts, only with the truncated mutant protein (Figure 4B, lanes 4–6). Conversely, immunoprecipitation of BRCA2 from EUFA423 B2 (Figure 4C, lanes 1–3) or EUFA423 (Figure 4C, lanes 4–6) cell extracts demonstrates that full-length BRCA2, as well as the cancer-associated $BRCA2^{9000insA}$ truncated mutant protein, interact with RNAPII detected by an antibody (601) against its CTD (Nojima et al., 2015). Reciprocally, this RNAPII antibody also co-immunoprecipitates full-length BRCA2 and the cancer-associated $BRCA2^{9000insA}$ truncated mutant protein (Figure 4D, lanes 1–6). RNAPII that co-immunoprecipitates with full-length or truncated mutant BRCA2 is phosphorylated on Ser2 and Ser5 residues in its CTD (Figures 4C and 4D). Taken together, our findings demonstrate that RNAPII and BRCA2 interact in human cells and that the cancer-associated $BRCA2^{9000insA}$ truncated mutant protein retains this interaction.

BRCA2 Inactivation Diminishes PAF1 Recruitment at PPP Sites

The regulated release of RNAPII from PPP sites requires the recruitment of PAF1 (Chen et al., 2015; Yu et al., 2015). Strikingly, ChIP analysis of PAF1 after BRCA2 depletion (Figure 5A) demonstrates reduced recruitment at both the PPP sites and across the transcription units of the actively transcribed *ACTB* and *GAPDH* genes in comparison with controls. A similar pattern of reduced PAF1 recruitment also occurs in *BRCA2*-deficient EUFA423 cells when compared with EUFA423 B2 control cells complemented with full-length BRCA2 in not only *ACTB* and *GAPDH* (Figure 5B) but also *TEFM*, *CALM3*, *VCL*, *RPL13A*, and *MRPL21* (Figures S3A–S3E), implicating the C-terminal region of BRCA2 spanning residues 3,254–3,418 that is absent from the truncated proteins encoded by the $BRCA2^{7691insAT / 9000insA}$ alleles in this process.

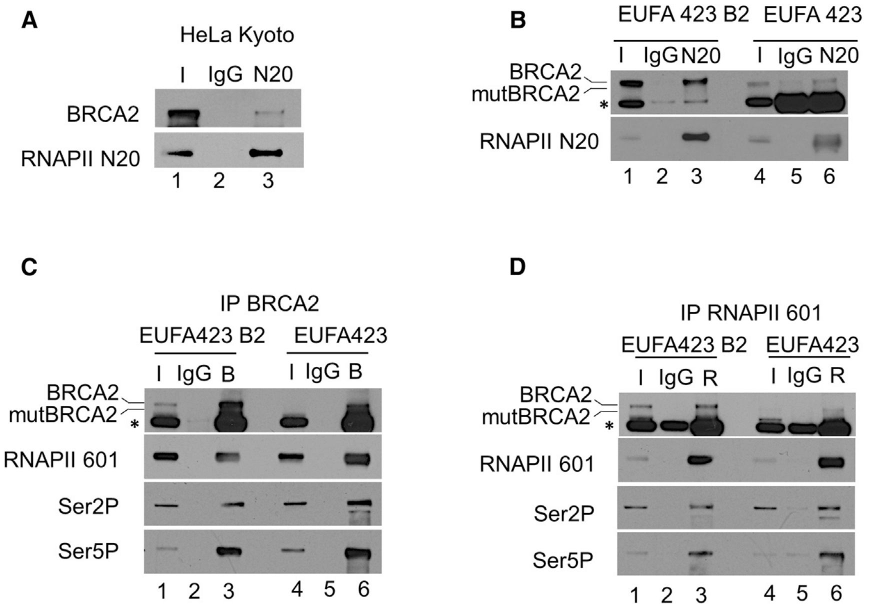

Figure 4. BRCA2 Interacts with RNAPII

(A) IP-western analysis showing the interaction between RNAPII and BRCA2 after IP with RNAPII N20 antibody in HeLa Kyoto cell extracts, followed by western blotting with the indicated antibodies.

(B) A similar analysis showing the interaction between RNAPII and BRCA2 in EUFA423 B2 and EUFA423 cells. Bars indicate the relative positions of the wild-type or truncated form of BRCA2 (mutBRCA2) encoded by *BRCA2*^{9000insA}.

(C) IP-western analysis with BRCA2 (Ab-1) antibody showing interaction with phosphorylated forms of RNAPII in EUFA423 B2 and EUFA423 cells.

(D) Reciprocal IP-western analysis with RNAPII antibody (601) showing interaction with BRCA2 and mutBRCA2. Inputs represent 10% of the total cell lysates.

Asterisk (*) denotes a nonspecific band.

BRCA2 Inactivation Decreases Histone H2B Ubiquitination

PAF1 recruitment to RNAPII at PPP sites in turn normally conscripts the RNF20/40 E3 ubiquitin ligase complex, which triggers nucleosome disassembly by ubiquitinating histone H2B on Lys (K)120, an alteration thought to facilitate transcription elongation by opening chromatin structure downstream of the active RNAPII holoenzyme (Van Oss et al., 2016; Wu et al., 2014; Xiao et al., 2005). The decreased recruitment of PAF1 to the *GAPDH* gene following *BRCA2* inactivation is accompanied by diminished histone H2B K120 ubiquitination detected by ChIP in both BRCA2-depleted cells (compared to controls) (Figure 5C) and EUFA423 cells (compared to EUFA423 B2 controls) (Figure 5D). Moreover, decreased PAF1 recruitment to RNAPII and reduced histone H2B K120 ubiquitination after BRCA2 depletion are associated with an overall reproducible and quantifiable decrease in the incorporation of 5-ethynyl uridine (EU) into nascent RNA detected by immunofluorescence staining (Figure S4), suggesting that transcription elongation is attenuated across the transcriptome.

PAF1 Overexpression Ameliorates R-Loop Accrual following BRCA2 Deficiency

These observations prompted us to test whether reduced PAF1 recruitment to RNAPII is a mechanism causing R-loop accrual at PPP sites in *BRCA2*-deficient cells. Indeed, depletion of PAF1 (Figure S1B) triggers R-loop accumulation at PPP sites of *GAPDH* detected by DRIP analysis, phenocopying the pattern induced by BRCA2 depletion (Figure 6A). Combined depletion of both BRCA2 and PAF1 (Figure S1B) does not significantly enhance this phenotype (Figure 6A), indicating that they are epistatic to one another and may thus operate via a common pathway. R-loop detection in these experiments is markedly reduced by RNase H (Figure 6A), validating its specificity. Conversely, overexpression of PAF1 in *BRCA2*-deficient EUFA423 cells (Figure 6B) ameliorates R-loop accumulation at PPP sites of *GAPDH* to a level resembling EUFA423 B2 controls (Figure 6C). Thus collectively, our results suggest that R-loop accrual at PPP sites of actively transcribed genes after BRCA2 inactivation is mediated by the reduced recruitment of PAF1 to paused RNAPII.

DISCUSSION

Our work suggests a model (Figure 6D) in which BRCA2 interacts with the RNAPII holoenzyme to regulate transcription elongation by augmenting the recruitment of PAF1 to the PPP sites of actively transcribed genes. BRCA2 inactivation diminishes PAF1 recruitment and may consequently reduce downstream chromatin disassembly that usually facilitates transcription elongation. These abnormalities cause RNAPII to accumulate at PPP sites, accompanied by the site-specific accrual of unscheduled R-loops that consequently lead to DNA breaks, via cleavage by the ERCC4 endonuclease (Sollier et al., 2014). Our findings suggest that R-loop formation and cleavage into DNA breaks may occur at multiple loci, raising the possibility that the mechanism we report here is a major source of genome damage following *BRCA2* inactivation.

Our model provides a mechanism for the observation that unscheduled R-loops accumulate after BRCA2 depletion (Bhatia et al., 2014), which has attracted much topical attention because of the established links between R-loop accrual and genomic instability (Aguilera and García-Muse, 2012; Hamperl and Cimprich, 2014). Indeed, we recently found that the structural chromosomal aberrations typical of BRCA2 deficiency, including radial chromosomes thought to signify aberrations in DNA repair by homologous recombination, are reduced by the overexpression of RNase H1 (Tan et al., 2017). When taken together with these findings, the work we report here speaks to the notion that unscheduled R-loop formation caused by defective transcription elongation is an underlying cause of genomic instability after BRCA2 inactivation. Indeed, we show here that

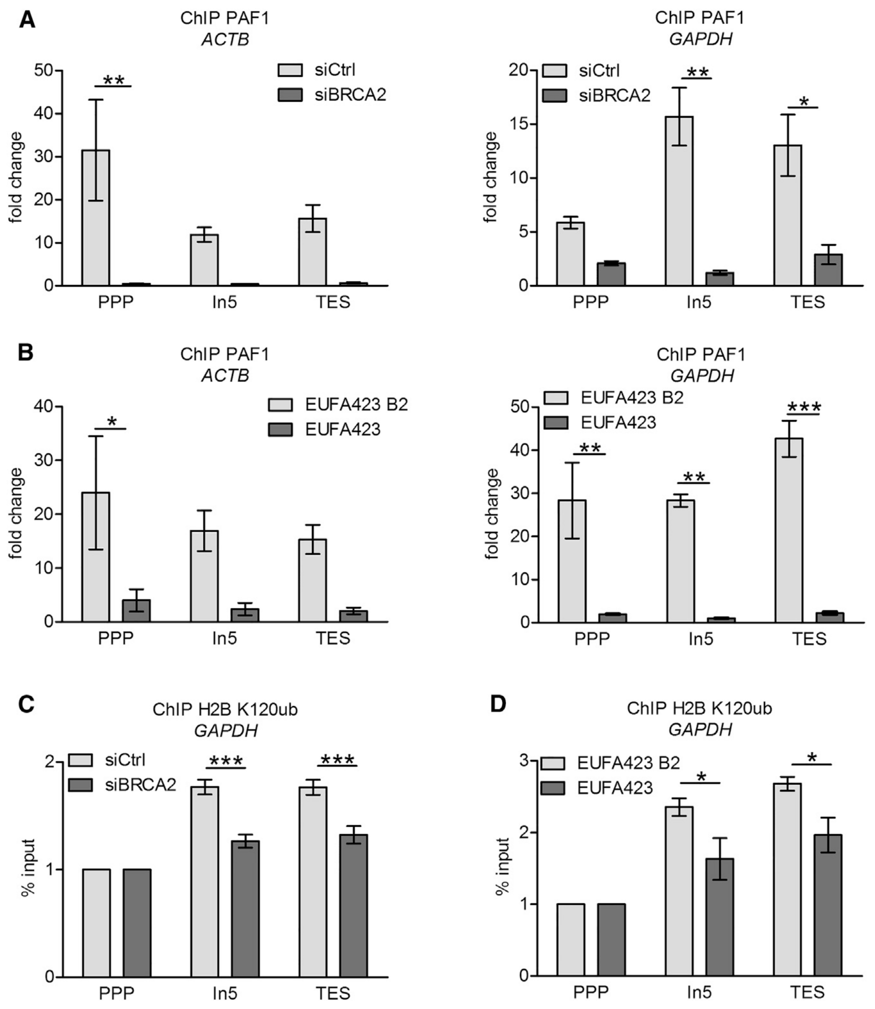

Figure 5. BRCA2 Inactivation Diminishes PAF1 Recruitment at PPP Sites and Subsequent H2B K120 Ubiquitination

(A and B) ChIP analyses with PAF1 antibody of the *ACTB* and *GAPDH* genes in HeLa Kyoto cells 72 hr after transfection with siCtrl or siBRCA2 (A) and EUFA423 B2 and EUFA423 cells (B).

(C and D) ChIP analyses with H2B K120ub antibody of *GAPDH* gene in HeLa Kyoto cells 72 hr after transfection with siCtrl or siBRCA2 cells (C) and EUFA423 B2 and EUFA423 cells (D). Fold change relative to the IgG isotype control antibody control is plotted.

Plots show the mean ± SEM from three independent experiments. Data were normalized to input (2%). The 2-way ANOVA test was performed for all pairwise comparisons to determine statistical significance. Statistically significant differences are indicated (*p < 0.05, **p < 0.01, and ***p < 0.001).

cancer-causing *BRCA2* mutations are sufficient to trigger such defects in the patient-derived EUFA423 cell line.

A small fraction of intracellular BRCA2 binds physically with BRCA1 (Chen et al., 1998), consistent with evidence that these tumor-suppressor proteins share at least certain biological functions during the cellular response to DNA damage (reviewed in Venkitaraman, 2014). However, the distribution and mechanism of R-loop accrual that we observe following BRCA2 inactivation appear to differ from that induced by depletion of BRCA1 or SETX. Our findings are consistent with prior reports (Hatchi et al., 2015; Zhang et al., 2017) in which siRNA-mediated BRCA1 depletion causes the accumulation of R-loops at 3′ sequences associated with the termination of transcription rather than at PPP sites. Consistent with these observations, such 3′ sites are enriched for mutations detected by next-generation sequencing in the genomes of BRCA1-deficient cancers (Hatchi et al., 2015). Puzzlingly, however, it has also been recently reported (Zhang et al., 2017) that R-loops accumulate at PPP sites in the mammary luminal epithelial cells of human *BRCA1* mutation carriers and that genetic ablation of NELF-B, a subunit of the negative elongation factor complex implicated in RNAPII pausing (Narita et al., 2003; Ye et al., 2001) that interacts with BRCA1, reduces mammary tumorigenesis in a genetically engineered murine model of *Brca1* deficiency (Nair et al., 2016). Thus, while the mechanisms responsible for R-loop accrual after BRCA1 inactivation remain unclear, we identify here a distinct function for BRCA2 in regulating transcription elongation by augmenting PAF1 recruitment to RNAPII at PPP sites.

Notably, cancer-associated truncating mutations that delete a C-terminal region of the BRCA2 protein retain RNAPII interaction but nevertheless diminish PAF1 recruitment to increase RNAPII accumulation and R-loop accrual at PPP sites. The C-terminal region of BRCA2 apparently necessary for PAF1 recruitment is often deleted in truncating mutations associated with breast and ovarian cancer (Rebbeck et al., 2015), suggesting its functional significance in tumor suppression. Thus, an unrecognized function of BRCA2 in regulating transcription elongation is subverted by cancer-causing mutations, in turn provoking the site-specific accumulation of genome-destabilizing R-loops, which can be cleaved by the ERCC4 endonuclease into DNA breaks. Our work suggests that unforeseen defects in transcription elongation—besides previously reported anomalies in DNA repair, replication, or mitosis—underlie the origins of genome instability and the pathogenesis of cancers in *BRCA2* mutation carriers.

EXPERIMENTAL PROCEDURES

Cell Culture

HeLa Kyoto (Adey et al., 2013), EUFA423 (Howlett et al., 2002), and EUFA423 B2 (stably transfected cell line with FLAG-BRCA2) (Hattori et al., 2011) cell lines were used in this study. HeLa Kyoto and EUFA423 cells were maintained in culture in DMEM containing 10% fetal bovine serum (FBS), 1% penicillin/streptomycin; for EUFA423 B2, G418 (0.75 mg/mL) was added to the medium.

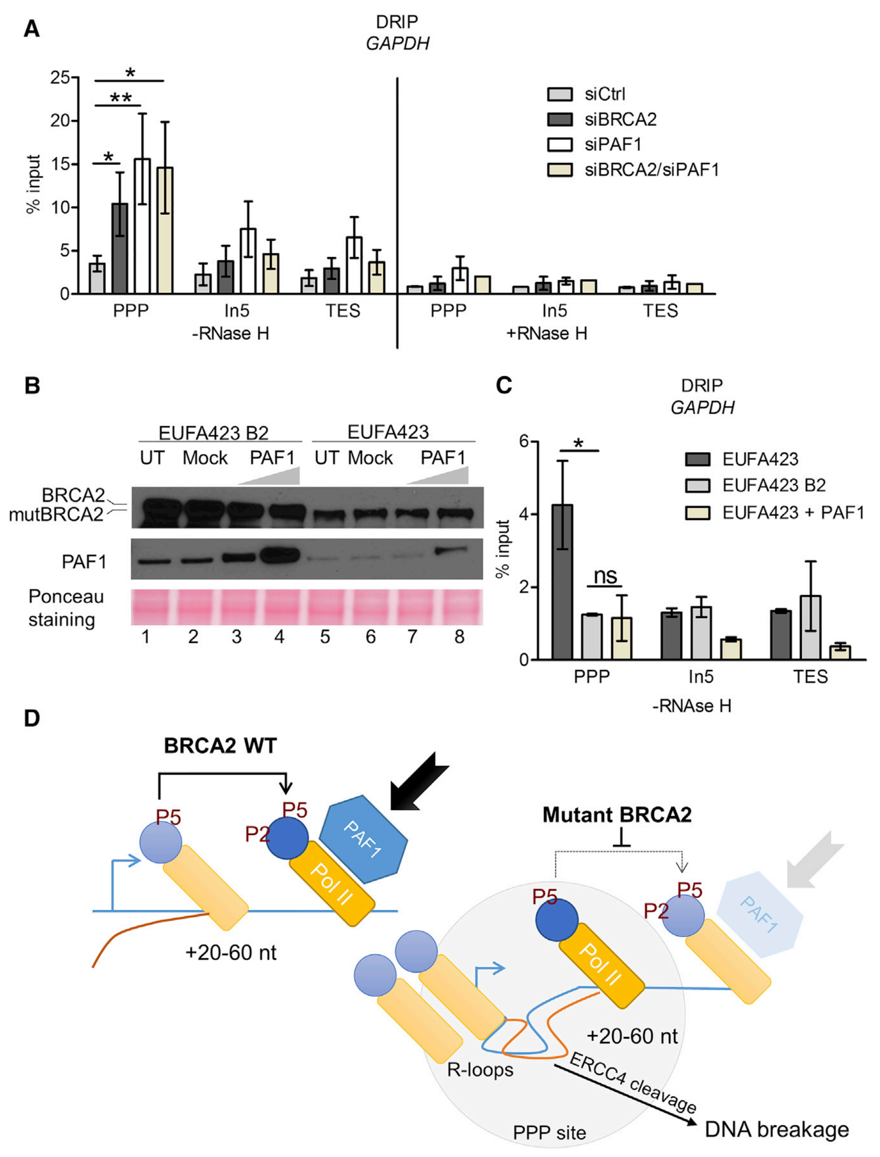

Figure 6. PAF1 Depletion Phenocopies, while PAF1 Overexpression Ameliorates, R-Loop Accumulation following BRCA2 Inactivation

(A) DRIP analysis with S9.6 antibody of *GAPDH* gene in HeLa Kyoto cells transfected with indicated siRNAs for 72 hr. R-loop dissolution by RNase H enzyme is shown as a control. Plots show the mean ± SEM from three independent experiments. The 2-way ANOVA test was performed for all pairwise comparisons to determine statistical significance. Statistically significant differences are indicated (*$p < 0.05$ and **$p < 0.01$).

(B) Western blot analysis of the overexpression of PAF1 in EUFA423 B2 and EUFA423 cells transfected with either 5 μg (lanes 3 and 7) or 10 μg (lanes 4 and 8) of the PAF1 construct. Cell lysates were analyzed 24 hr after transfection. Bars indicate the relative positions of the wild-type and truncated forms of BRCA2 (mutBRCA2).

(C) DRIP analysis of *GAPDH* gene (as in A) showing R-loop reduction after PAF1 overexpression for 24 hr in EUFA423 cells compared to untransfected EUFA423 or EUFA423 B2 controls. Plots show the mean ± SEM from three independent experiments. The 2-way ANOVA test was performed for all pairwise comparisons to determine statistical significance. Statistically significant differences are indicated (*$p < 0.05$; ns, not significant).

(D) Schematic model depicting that BRCA2 regulates transcription elongation by RNA polymerase II to prevent R-loop accumulation. Normally, BRCA2 augments the recruitment of PAF1 to RNAPII paused at PPP sites located ~20–60 nt from transcription start sites (arrow), fostering the switch to productive transcription elongation (left). This function is subverted by cancer-causing *BRCA2* mutations (right), diminishing PAF1 recruitment. Consequently, RNAPII accumulates at PPP sites, triggering the site-specific accrual of unscheduled R-loops, which are cleaved by the ERCC4 endonuclease to generate DNA breaks, triggering genomic instability and carcinogenesis following BRCA2 inactivation.

Cell Lysates, Immunoprecipitation, and Western Blotting

Total cell lysates (TCLs) were prepared from HeLa Kyoto, EUFA423, and EUFA423 B2 cells using NP40 lysis buffer (Hattori et al., 2011) supplemented with NaCl (final concentration, 500 mM) and Benzonase (350 μ/mL).

Immunoprecipitations (IPs) were performed using TCLs (0.25–0.5 mg) that were precleared with immunoglobulin G (IgG) and protein A/G beads prior to incubation with either BRCA2 (Ab-1) or RNAPII antibody (N20 or 601) overnight at 4°C. IPs were collected after incubation with Protein A/G Dynabeads and washed extensively with Tris buffered saline-0.1% Tween20 (TBST) and Tris-buffered saline (TBS). The IP targets were identified by western blotting on 4%–12% Bis-Tris/2-(N-morpholino)ethanesulfonic acid (BT/MES) SDS-PAGE/transfer overnight on polyvinylidene fluoride (PVDF) Immobilon membranes, blocking/primary antibody (overnight 4°C)/secondary antibody in TBST/5% milk and enhanced chemiluminescence (ECL)/ECL prime chemiluminescence detection.

Antibodies

Antibodies used in this study are listed in Table S1.

S9.6 antibody, used to detect RNA-DNA hybrids, was purified from the mouse hybridoma cell line S9.6 (ATCC- HB8730). Briefly, cell cultures were grown for ~7 days before supernatants were centrifuged at 1,500 × *g* for 10 minutes and 0.45-μm filter sterilized. Fractions containing high concentrations of antibody were dialyzed in 2 L PBS 1× overnight followed by a second dialysis in 500 mL PBS 1×/ 50% glycerol for at least 5 hr using an ÄKTA Avant purifier (GE Healthcare). The antibody was then diluted to a final concentration of 1 μg/μL and stored at −80°C.

Plasmid Transfection

JetPRIME transfection reagent from Polyplus was used according to manufacturer's instructions for transfecting per 15cm dish 5 or 10 μg pcDNA3_PAF1 (a gift from Dr. Matthew Meyerson, Dana-Farber Cancer Institute, Boston, MA, USA; Addgene plasmid 11059) (Rozenblatt-Rosen et al., 2005) as indicated.

siRNA Transfection

siRNAs were transfected using a standard calcium phosphate transfection protocol (Lossaint et al., 2013). siRNAs used were Negative Control siRNA (1027310, QIAGEN), human BRCA2 (ON-TARGET plus SMARTpool, L-003462-00, Dharmacon), human BRCA1 (5′-GGAACCUGUCUCCACAAAG-3′), human ERCC4 (ON-TARGET plus SMARTpool, L-019946-00-0005, Dharmacon), and human PAF1 (ON-TARGET plus SMARTpool, M-020349-01, Dharmacon).

ChIP

ChIP was performed as described previously (Hatchi et al., 2015). Briefly, cells were crosslinked in DMEM containing 1% formaldehyde (Sigma, F8775) at room temperature for 10 min with rotation. The crosslinking reaction was stopped by adding glycine (Sigma, G8898) to a final concentration of 0.125 M for 5 min at room temperature. After two washes with PBS, cells were collected in 5 mL PBS and centrifuged at 1,500 rpm for 5 min. Cell pellets were lysed in 5 mM EDTA, 50 mM Tris-HCl (pH 8.0), and 1% SDS supplemented with protease and phosphatase inhibitors. Total cell lysates were sonicated (Bioruptor, Diagenode) to obtain chromatin fragments of an average length of 200–800 bp and then centrifuged at 10,000 rpm for 10 min at 4°C. 10 μg chromatin was diluted 10-fold with 5 mM EDTA, 50 mM Tris-HCl (pH 8.0), 0.5% NP-40, and 200 mM NaCl supplemented with protease and phosphatase inhibitors. Chromatin extracts were precleared with protein G Dynabeads (Thermo Fisher) for 40 min. The precleared chromatin was incubated overnight with gentle rotation with antibodies or control antibody. The following day, antibodies were captured by adding 20uL of beads and incubated at 4°C with rotation for 2 hr. Immunoprecipitates were washed with: 1×: 20 mM Tris-HCl (pH 8), 2 mM EDTA, 0.1% SDS, 1% Triton X-100, and 165 mM NaCl; 1×: 20 mM Tris-HCl (pH 8), 2 mM EDTA, 0.1% SDS, 1% Triton X-100, and 500 mM NaCl; 1×: 10 mM Tris-HCl (pH 8), 1 mM EDTA, 1% NP-40, 1% Na-deoxycholate, and 250 mM LiCl; 1×: 50 mM HEPES (pH 7.6), 1 mM EDTA, 1% NP-40, 0.7% Na-deoxycholate, and 500 mM LiCl; 2× with Tris-EDTA (TE) (10 mM Tris-HCl [pH7.5] and 1 mM EDTA). After the final wash, DNA was eluted with 150 μL of 0.1 M sodium carbonate, 1% SDS at 65°C for 30 min with 1,200 rpm agitation. To reverse crosslink, 6 μL of 5 M NaCl and 2 μL proteinase K (20 mg/mL) were added and incubated at 65°C with gentle agitation for at least 3 hr. DNA was purified using a PCR purification kit (QIAGEN) and analyzed by qPCR using SYBR green mix (Roche) with the primers of interest. For γH2AX ChIP, the values were normalized to EUFA423 B2.

DRIP

Genomic DNA, extracted with a previously described phenol/chloroform procedure (Bhatia et al., 2014; Ginno et al., 2012), was digested at 37°C with a cocktail of restriction enzymes (20 U/L EcoRI, 20 U HindIII, 20 U XbaI, 25 U SSPI, and 10 U BsrGI per 50μg DNA) in buffer 2.1 (NEB) with or without RNase H (5 U/μL, NEB). Following a second DNA purification step with standard phenol/chloroform procedure, 10 μg of digested DNA was diluted in 900 μL of TE buffer (5 mM EDTA and 50 mM Tris HCl [pH 8]) and 100 μL of 10X DRIP buffer (100 mM NaH_2PO_4, 1.4 M NaCl, and 0.5% Triton-X) was added. Chromatin was precleared with 20 μL protein G Dynabeads for 40 min at 4°C. Then, 10 μg S9.6 antibody was added to the supernatant and incubated at 4°C overnight with gentle rotation. Antibody capture was done with 20 μL beads and incubation at 4°C for 2 hr. Immunoprecipitates were washed twice with 1× DRIP buffer, and once in 1× DRIP buffer + 330 mM NaCl. After the last wash, DNA was eluted by adding DRIP elution buffer (50 mM Tris-HCl [pH 8], 10 mM EDTA, and 0.5% SDS) and incubated at 65°C for 45 min. DNA was finally purified using PCR purification kit (QIAGEN) and analyzed by qPCR with SYBR green mix (Roche).

Primers

Primers used in this study are listed in Table S2.

Immunofluorescence for R-Loop Detection

The appropriate number of cells was plated on coverslips the day before transfection and after transfection fixed in cold methanol for 10 min at −20°C, followed by incubation for 1 min in cold acetone at room temperature. The coverslips were then quickly washed in saline sodium citrate (SSC) 4× buffer thrice and then incubated for 30 min in SSC 4×, 3% BSA to prevent nonspecific interaction. Primary antibodies were incubated overnight at 4°C (S9.6 1/100, purified in house). After three washes with PBS-tween 0.05%, coverslips were incubated at room temperature with secondary antibodies Alexa 488 and Alexa Fluor 568 (Molecular Probes, 1/500). Coverslips were mounted with DAKO mounting medium (S3023) supplemented with DAPI (1.5 μg/mL, D9542, Sigma) after three additional washes with PBS-tween 0.05%. Stained cells were imaged on a Zeiss 880 confocal microscope. Maximum projections of the z stacks of each field were analyzed using Fiji software (https://fiji.sc/). Intensity was calculated using a DAPI mask.

EU Incorporation and IF

Cells were treated with a modified nucleotide (EU; E10345) at 2 mM concentration for 30 min prior to fixation (Jao and Salic, 2008) and detection with the Click-iT RNA Alexa Fluor 594 Imaging Kit (C10330, Thermo Fisher) according to the manufacturer's instructions. Image acquisition and analysis were performed as described above.

Statistical Analysis

Statistical significance was determined using a two-tailed Student's t test for testing significance between two groups. For ChIP and DRIP analysis, statistical significances were performed using two-way analysis of variance (2-way ANOVA) followed by a Bonferroni correction. All pairwise comparisons were tested, but only statistically significant differences are indicated with asterisks in the figures. All tests were performed using GraphPad Prism 5.0. See Table S3 for more information.

SUPPLEMENTAL INFORMATION

Supplemental Information includes four figures and three tables and can be found with this article online at https://doi.org/10.1016/j.celrep.2017.12.086.

ACKNOWLEDGMENTS

We thank Dr. Matthew Meyerson (Dana-Farber Cancer Institute, Boston) for the gift of pcDNA3_PAF1 plasmid and members of A.R.V.'s laboratory for helpful comments and discussion. This work was supported by Medical Research Council programme grants MC_UU_12022/1 and MC_UU_12022/8 (to A.R.V.).

AUTHOR CONTRIBUTIONS

A.R.V. and M.K.K.S. conceived the project. X.R. performed experiments, analyzed data, and prepared Figures 1C–1E, 2, 3, 4, 5, and 6; M.K.K.S. performed experiments, analyzed data, and prepared Figure 4; Ç.H.W. performed experiments, analyzed data (Figures 2E, 6A, and 6B), and prepared Figure 6B. M.K.K.S. and X.R. are joint first authors listed in arbitrary order. A.R.V. supervised the work, analyzed data, and wrote the manuscript with assistance from the other authors.

DECLARATION OF INTERESTS

The authors declare no competing interests.

Received: August 15, 2017
Revised: November 9, 2017
Accepted: December 22, 2017
Published: January 23, 2018

REFERENCES

Adelman, K., and Lis, J.T. (2012). Promoter-proximal pausing of RNA polymerase II: emerging roles in metazoans. Nat. Rev. Genet. *13*, 720–731.

Adey, A., Burton, J.N., Kitzman, J.O., Hiatt, J.B., Lewis, A.P., Martin, B.K., Qiu, R., Lee, C., and Shendure, J. (2013). The haplotype-resolved genome and epigenome of the aneuploid HeLa cancer cell line. Nature *500*, 207–211.

Aguilera, A., and García-Muse, T. (2012). R loops: from transcription byproducts to threats to genome stability. Mol. Cell *46*, 115–124.

Bhatia, V., Barroso, S.I., García-Rubio, M.L., Tumini, E., Herrera-Moyano, E., and Aguilera, A. (2014). BRCA2 prevents R-loop accumulation and associates with TREX-2 mRNA export factor PCID2. Nature *511*, 362–365.

Boehm, A.K., Saunders, A., Werner, J., and Lis, J.T. (2003). Transcription factor and polymerase recruitment, modification, and movement on dhsp70 in vivo in the minutes following heat shock. Mol. Cell. Biol. *23*, 7628–7637.

Breast Cancer Linkage Consortium (1999). Cancer risks in BRCA2 mutation carriers. J. Natl. Cancer Inst. *91*, 1310–1316.

Chapman, R.D., Heidemann, M., Albert, T.K., Mailhammer, R., Flatley, A., Meisterernst, M., Kremmer, E., and Eick, D. (2007). Transcribing RNA polymerase II is phosphorylated at CTD residue serine-7. Science *318*, 1780–1782.

Chen, J., Silver, D.P., Walpita, D., Cantor, S.B., Gazdar, A.F., Tomlinson, G., Couch, F.J., Weber, B.L., Ashley, T., Livingston, D.M., and Scully, R. (1998). Stable interaction between the products of the BRCA1 and BRCA2 tumor suppressor genes in mitotic and meiotic cells. Mol. Cell *2*, 317–328.

Chen, F.X., Woodfin, A.R., Gardini, A., Rickels, R.A., Marshall, S.A., Smith, E.R., Shiekhattar, R., and Shilatifard, A. (2015). PAF1, a molecular regulator of promoter-proximal pausing by RNA polymerase II. Cell *162*, 1003–1015.

Cheng, C., and Sharp, P.A. (2003). RNA polymerase II accumulation in the promoter-proximal region of the dihydrofolate reductase and gamma-actin genes. Mol. Cell. Biol. *23*, 1961–1967.

Cho, E.J., Kobor, M.S., Kim, M., Greenblatt, J., and Buratowski, S. (2001). Opposing effects of Ctk1 kinase and Fcp1 phosphatase at Ser 2 of the RNA polymerase II C-terminal domain. Genes Dev. *15*, 3319–3329.

Ginno, P.A., Lott, P.L., Christensen, H.C., Korf, I., and Chédin, F. (2012). R-loop formation is a distinctive characteristic of unmethylated human CpG island promoters. Mol. Cell *45*, 814–825.

Hamperl, S., and Cimprich, K.A. (2014). The contribution of co-transcriptional RNA:DNA hybrid structures to DNA damage and genome instability. DNA Repair (Amst.) *19*, 84–94.

Hatchi, E., Skourti-Stathaki, K., Ventz, S., Pinello, L., Yen, A., Kamieniarz-Gdula, K., Dimitrov, S., Pathania, S., McKinney, K.M., Eaton, M.L., et al. (2015). BRCA1 recruitment to transcriptional pause sites is required for R-loop-driven DNA damage repair. Mol. Cell *57*, 636–647.

Hattori, H., Skoulidis, F., Russell, P., and Venkitaraman, A.R. (2011). Context dependence of checkpoint kinase 1 as a therapeutic target for pancreatic cancers deficient in the BRCA2 tumor suppressor. Mol. Cancer Ther. *10*, 670–678.

Howlett, N.G., Taniguchi, T., Olson, S., Cox, B., Waisfisz, Q., De Die-Smulders, C., Persky, N., Grompe, M., Joenje, H., Pals, G., et al. (2002). Biallelic inactivation of BRCA2 in Fanconi anemia. Science *297*, 606–609.

Jao, C.Y., and Salic, A. (2008). Exploring RNA transcription and turnover in vivo by using click chemistry. Proc. Natl. Acad. Sci. USA *105*, 15779–15784.

Jonkers, I., and Lis, J.T. (2015). Getting up to speed with transcription elongation by RNA polymerase II. Nat. Rev. Mol. Cell Biol. *16*, 167–177.

Krumm, A., Hickey, L.B., and Groudine, M. (1995). Promoter-proximal pausing of RNA polymerase II defines a general rate-limiting step after transcription initiation. Genes Dev. *9*, 559–572.

Lossaint, G., Larroque, M., Ribeyre, C., Bec, N., Larroque, C., Décaillet, C., Gari, K., and Constantinou, A. (2013). FANCD2 binds MCM proteins and controls replisome function upon activation of s phase checkpoint signaling. Mol. Cell *51*, 678–690.

Marshall, N.F., Peng, J., Xie, Z., and Price, D.H. (1996). Control of RNA polymerase II elongation potential by a novel carboxyl-terminal domain kinase. J. Biol. Chem. *271*, 27176–27183.

Nair, S.J., Zhang, X., Chiang, H.C., Jahid, M.J., Wang, Y., Garza, P., April, C., Salathia, N., Banerjee, T., Alenazi, F.S., et al. (2016). Genetic suppression reveals DNA repair-independent antagonism between BRCA1 and COBRA1 in mammary gland development. Nat. Commun. *7*, 10913.

Narita, T., Yamaguchi, Y., Yano, K., Sugimoto, S., Chanarat, S., Wada, T., Kim, D.K., Hasegawa, J., Omori, M., Inukai, N., et al. (2003). Human transcription elongation factor NELF: identification of novel subunits and reconstitution of the functionally active complex. Mol. Cell. Biol. *23*, 1863–1873.

Nojima, T., Gomes, T., Grosso, A.R.F., Kimura, H., Dye, M.J., Dhir, S., Carmo-Fonseca, M., and Proudfoot, N.J. (2015). Mammalian NET-Seq reveals genome-wide nascent transcription coupled to RNA processing. Cell *161*, 526–540.

Rasmussen, E.B., and Lis, J.T. (1993). In vivo transcriptional pausing and cap formation on three Drosophila heat shock genes. Proc. Natl. Acad. Sci. USA *90*, 7923–7927.

Rebbeck, T.R., Mitra, N., Wan, F., Sinilnikova, O.M., Healey, S., McGuffog, L., Mazoyer, S., Chenevix-Trench, G., Easton, D.F., Antoniou, A.C., et al.; CIMBA Consortium (2015). Association of type and location of BRCA1 and BRCA2 mutations with risk of breast and ovarian cancer. JAMA *313*, 1347–1361.

Rougvie, A.E., and Lis, J.T. (1988). The RNA polymerase II molecule at the 5′ end of the uninduced hsp70 gene of D. melanogaster is transcriptionally engaged. Cell *54*, 795–804.

Rozenblatt-Rosen, O., Hughes, C.M., Nannepaga, S.J., Shanmugam, K.S., Copeland, T.D., Guszczynski, T., Resau, J.H., and Meyerson, M. (2005). The parafibromin tumor suppressor protein is part of a human Paf1 complex. Mol. Cell. Biol. *25*, 612–620.

Sanz, L.A., Hartono, S.R., Lim, Y.W., Steyaert, S., Rajpurkar, A., Ginno, P.A., Xu, X., and Chédin, F. (2016). Prevalent, dynamic, and conserved R-loop structures associate with specific epigenomic signatures in mammals. Mol. Cell *63*, 167–178.

Schlegel, B.P., Green, V.J., Ladias, J.A., and Parvin, J.D. (2000). BRCA1 interaction with RNA polymerase II reveals a role for hRPB2 and hRPB10alpha in activated transcription. Proc. Natl. Acad. Sci. USA *97*, 3148–3153.

Scully, R., Anderson, S.F., Chao, D.M., Wei, W., Ye, L., Young, R.A., Livingston, D.M., and Parvin, J.D. (1997). BRCA1 is a component of the RNA polymerase II holoenzyme. Proc. Natl. Acad. Sci. USA *94*, 5605–5610.

Sijbers, A.M., de Laat, W.L., Ariza, R.R., Biggerstaff, M., Wei, Y.F., Moggs, J.G., Carter, K.C., Shell, B.K., Evans, E., de Jong, M.C., et al. (1996). Xeroderma pigmentosum group F caused by a defect in a structure-specific DNA repair endonuclease. Cell *86*, 811–822.

Skourti-Stathaki, K., Proudfoot, N.J., and Gromak, N. (2011). Human senataxin resolves RNA/DNA hybrids formed at transcriptional pause sites to promote Xrn2-dependent termination. Mol. Cell *42*, 794–805.

Sollier, J., and Cimprich, K.A. (2015). Breaking bad: R-loops and genome integrity. Trends Cell Biol. *25*, 514–522.

Sollier, J., Stork, C.T., García-Rubio, M.L., Paulsen, R.D., Aguilera, A., and Cimprich, K.A. (2014). Transcription-coupled nucleotide excision repair factors promote R-loop-induced genome instability. Mol. Cell *56*, 777–785.

Tan, S.L.W., Chadha, S., Liu, Y., Gabasova, E., Perera, D., Ahmed, K., Constantinou, S., Renaudin, X., Lee, M., Aebersold, R., et al. (2017). A class of environmental and endogenous toxins induces BRCA2 haploinsufficiency and genome instability. Cell *169*, 1105–1118.

Van Oss, S.B., Shirra, M.K., Bataille, A.R., Wier, A.D., Yen, K., Vinayachandran, V., Byeon, I.L., Cucinotta, C.E., Héroux, A., Jeon, J., et al. (2016). The histone modification domain of Paf1 complex subunit Rtf1 directly stimulates H2B ubiquitylation through an interaction with Rad6. Mol. Cell *64*, 815–825.

Venkitaraman, A.R. (2014). Cancer suppression by the chromosome custodians, BRCA1 and BRCA2. Science *343*, 1470–1475.

Wu, L., Li, L., Zhou, B., Qin, Z., and Dou, Y. (2014). H2B ubiquitylation promotes RNA Pol II processivity via PAF1 and pTEFb. Mol. Cell *54*, 920–931.

Xiao, T., Kao, C.F., Krogan, N.J., Sun, Z.W., Greenblatt, J.F., Osley, M.A., and Strahl, B.D. (2005). Histone H2B ubiquitylation is associated with elongating RNA polymerase II. Mol. Cell. Biol. *25*, 637–651.

Ye, Q., Hu, Y.F., Zhong, H., Nye, A.C., Belmont, A.S., and Li, R. (2001). BRCA1-induced large-scale chromatin unfolding and allele-specific effects of cancer-predisposing mutations. J. Cell Biol. *155*, 911–921.

Yu, M., Yang, W., Ni, T., Tang, Z., Nakadai, T., Zhu, J., and Roeder, R.G. (2015). RNA polymerase II-associated factor 1 regulates the release and phosphorylation of paused RNA polymerase II. Science *350*, 1383–1386.

Zhang, X., Chiang, H.C., Wang, Y., Zhang, C., Smith, S., Zhao, X., Nair, S.J., Michalek, J., Jatoi, I., Lautner, M., et al. (2017). Attenuation of RNA polymerase II pausing mitigates BRCA1-associated R-loop accumulation and tumorigenesis. Nat. Commun. *8*, 15908.

Zhao, D.Y., Gish, G., Braunschweig, U., Li, Y., Ni, Z., Schmitges, F.W., Zhong, G., Liu, K., Li, W., Moffat, J., et al. (2016). SMN and symmetric arginine dimethylation of RNA polymerase II C-terminal domain control termination. Nature *529*, 48–53.

Cell Reports, Volume *22*

Supplemental Information

BRCA2 Regulates Transcription Elongation by
RNA Polymerase II to Prevent R-Loop Accumulation

Mahmud K.K. Shivji, Xavier Renaudin, Çiğdem H. Williams, and Ashok R. Venkitaraman

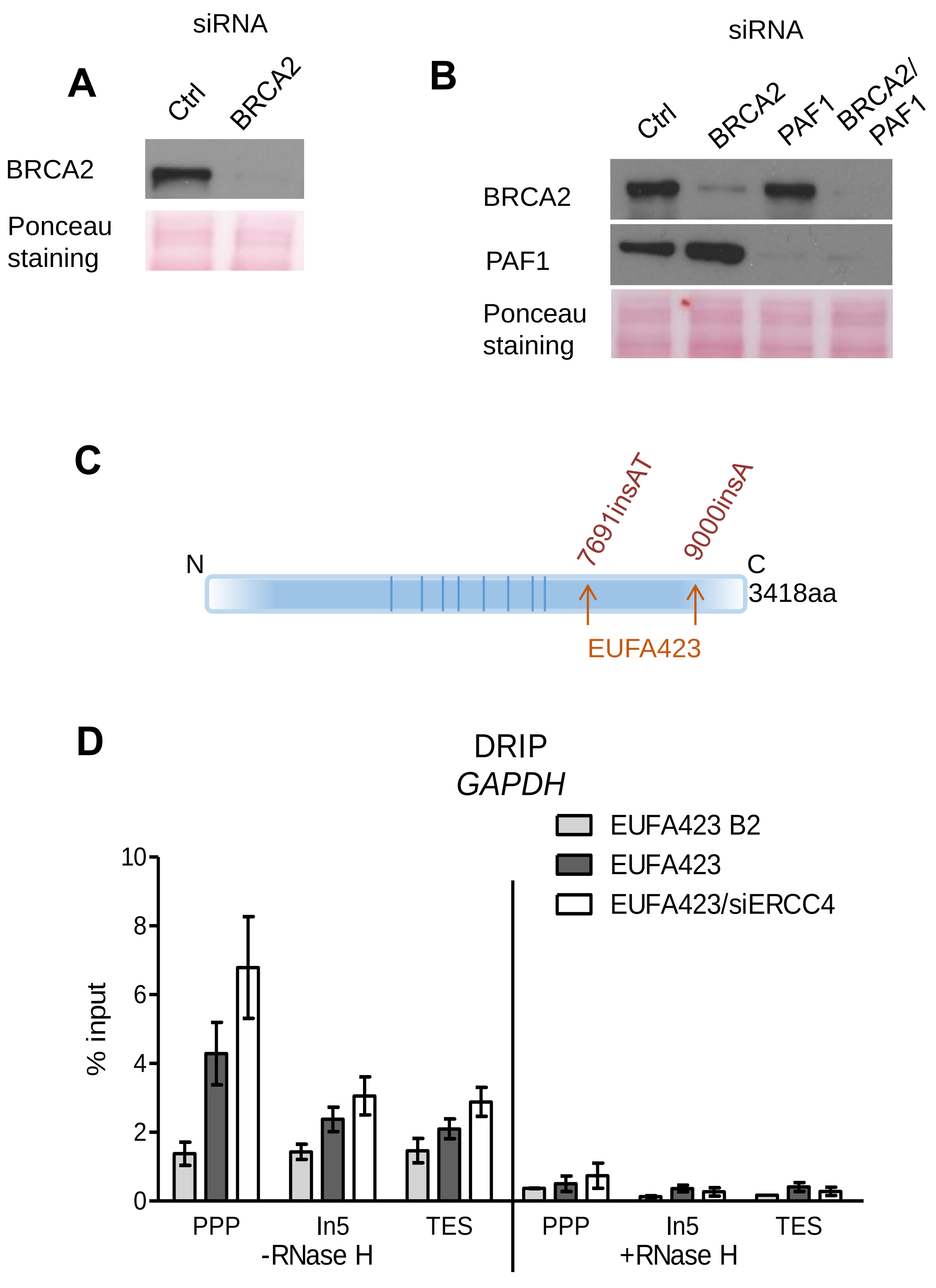

Figure S1. Related to Figures 1, 2 and 6.

(**A**)(**B**) Western blots for BRCA2 or PAF1 in HeLa Kyoto cells treated with the indicated siRNAs. (**C**) Schematic showing the location in the 3418aa human BRCA2 protein of the truncating mutations *BRCA2*^{7691insAT} and *BRCA2*^{9000insA} present in patient-derived EUFA423 cells. EUFA423 B2 control cells have been reconstituted with full-length wild-type BRCA2. (**D**) DRIP analyses with S9.6 antibody of the *GAPDH* gene in EUFA423 B2 and EUFA423 cells, without or with siRNA targeting ERCC4. R-loop digestion by RNAse H enzyme is shown as a control. Plots show the mean±s.e.m from two independent experiments.

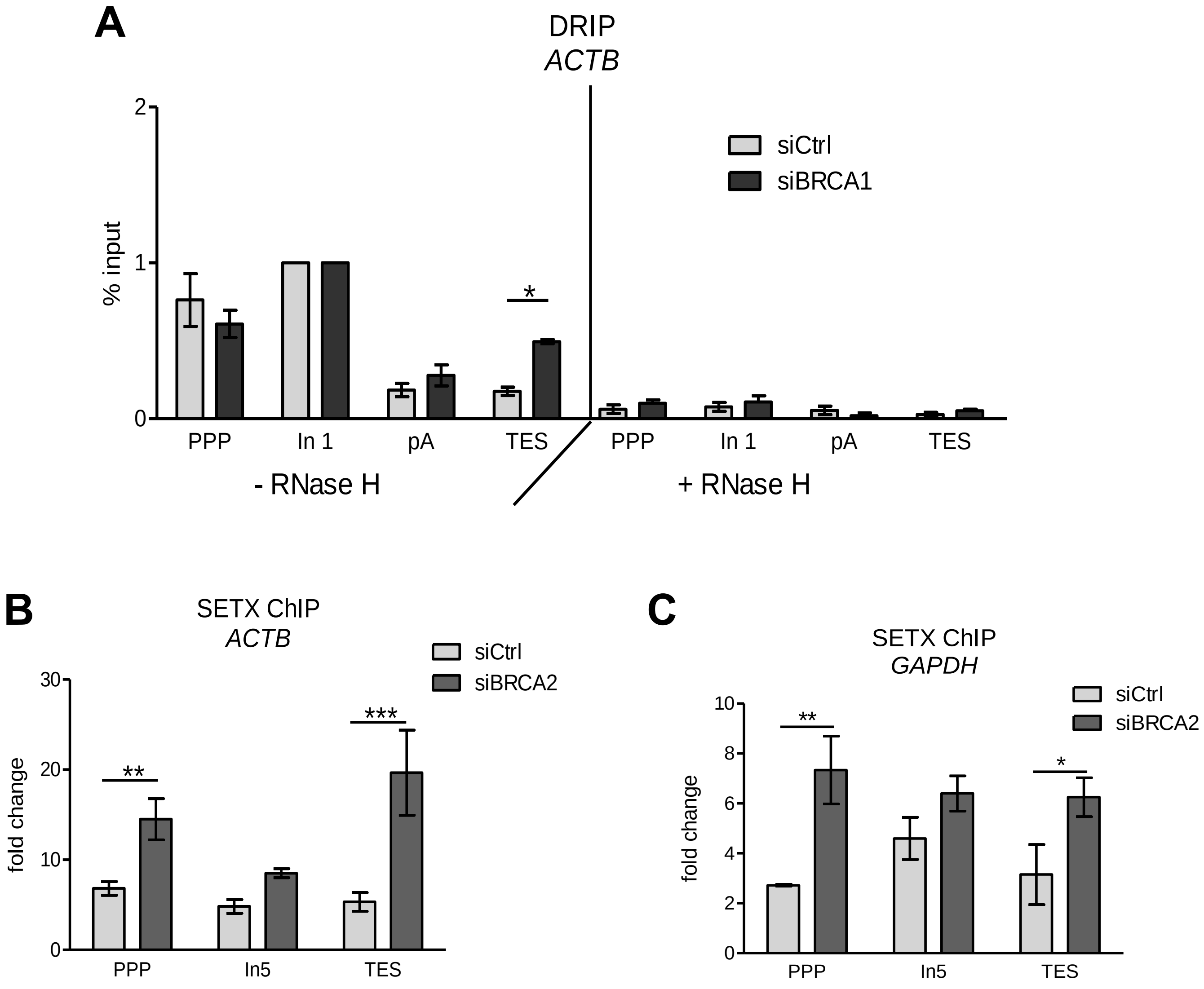

Figure S2. Related to Figure 2.

(**A**) DRIP analysis with S9.6 antibody of ACTB gene in HeLa Kyoto cells transfected with indicated siCtrl or siBRCA1 for 72h. R-loop dissolution by RNAse H enzyme is shown as a control. Error bars indicate the mean±s.e.m from three independent experiments. The 2-way ANOVA test was performed for all pairwise comparisons to determine statistical significance. Statistically significant differences are indicated. Data are normalized to In1. (**B**) ChIP analysis with SETX antibody of *ACTB* gene, and (**C**) *GAPDH* gene in HeLa Kyoto cells transfected with siCtrl or siBRCA2 for 72h. Error bars indicate the mean±s.e.m of three independent experiments. The 2-way ANOVA test was performed for all pairwise comparisons to determine statistical significance. Statistically significant differences are indicated *p<0.05, **p<0.01, ***p<0.001.

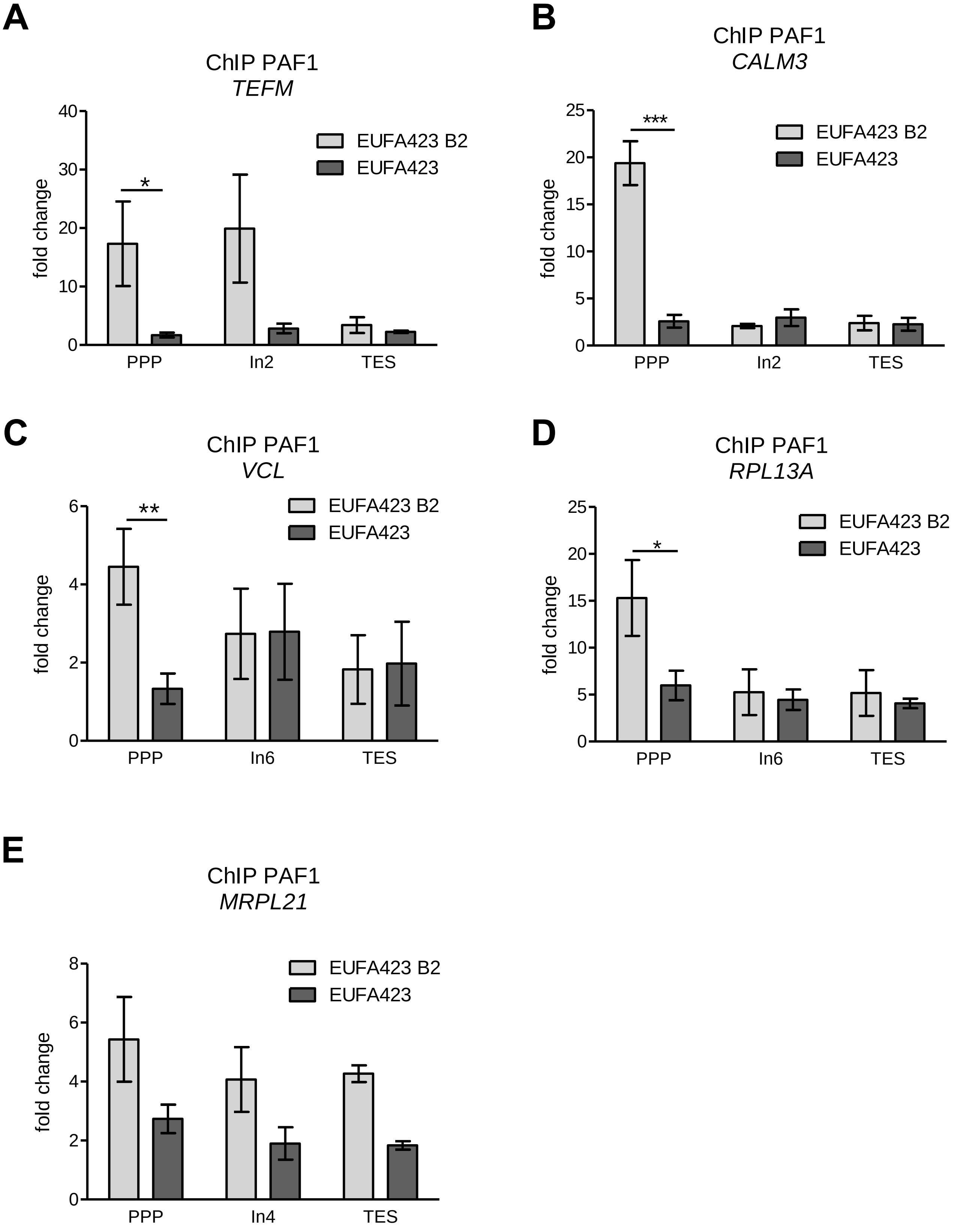

Figure S3. Related to Figure 5.

ChIP analyses of (**A**) *TEFM*, (**B**) *CALM3*, (**C**) *VCL*, (**D**) *RPL13A* and (**E**) *MRPL21* genes with PAF1 antibody in EUFA423 B2 and EUFA423 cells. The 2-way ANOVA test was performed for all pairwise comparisons to determine statistical significance. Statistically significant differences are indicated *p<0.05, **p<0.01, ***p<0.001.

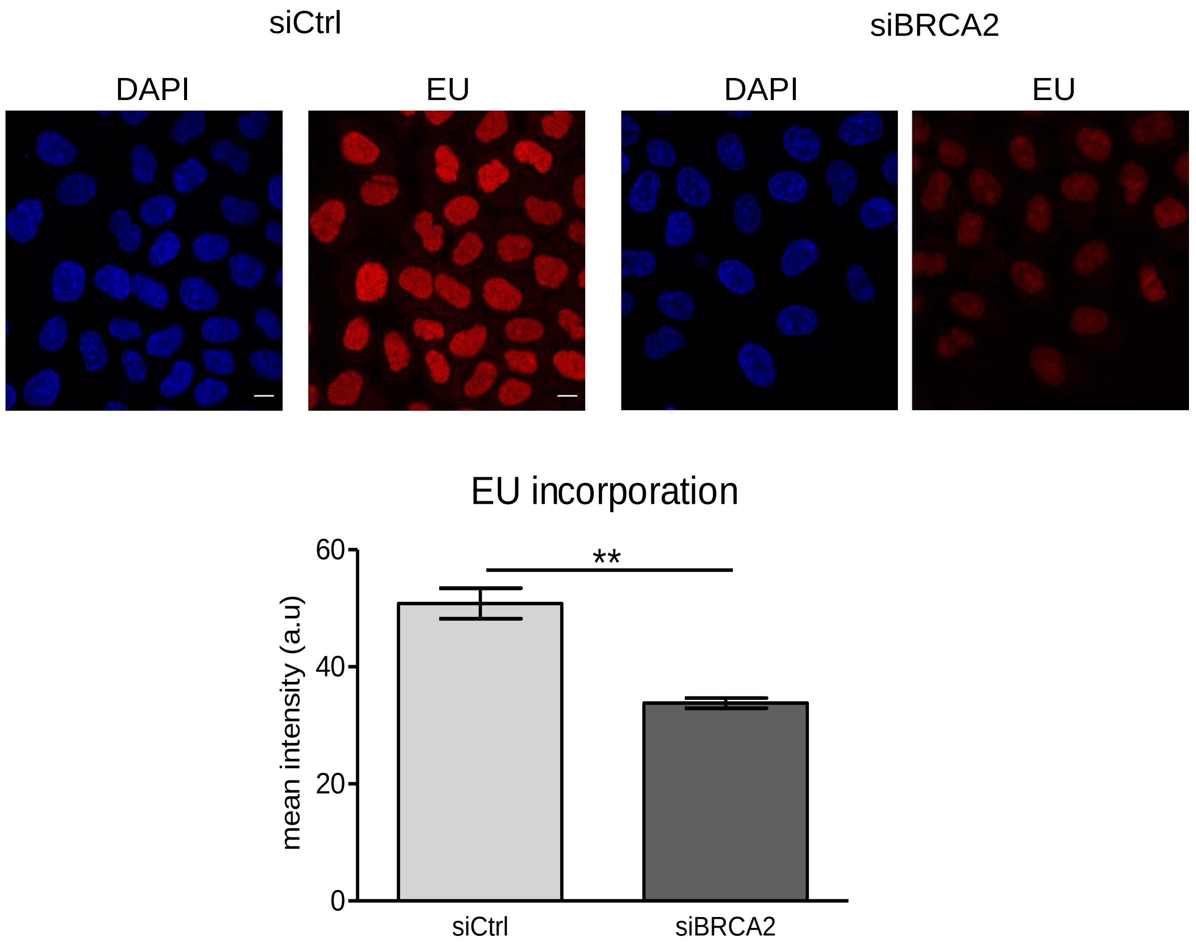

Figure S4. Related to Figure 5.

Immunofluorescence of siCtrl or siBRCA2 transfected HeLa Kyoto cells labelled with the nucleotide analogue 5-Ethynyl Uridine (EU) for 30 minutes to capture nascent RNA synthesis. Scale bars 10µm. The decrease in EU incorporation was quantitated in three independent experiments, and plotted in the bar graph as mean±s.e.m. The two-tailed Student's t-test was performed to determine statistical significance between the two groups. ** indicates $p<0.01$.

Supplemental Table S1. Related to Figures 1-6. Antibodies used in this study.

Antibody name	Clone No:	Cat. No:	Supplier	Stock Conc mg/ml	Application	Host	Clonality
BRCA2	Ab-1	OP95	Merck		WB/IP/ChIP	Mouse	mAb
H2B K120 Ubiquitin	D11	5546S	Cell Signalling		WB/ChIP	Mouse	mAb
H2B		Ab1790	Abcam		WB	Rabbit	pAb
γH2AX (phospho S139)		Ab2893	Abcam		ChIP	Rabbit	pAb
PAF1		Ab137519	Abcam		WB	Rabbit	pAb
PAF1		A300-173A	Bethyl	1ug/uL	ChIP	Rabbit	pAb
RNA polymerase II	MABI0601	MABI0601	MBL Itl	1ug/uL	ChIP	Mouse	mAb
RNA polymerase II **DISCONTINUED**	N20	SC-899	Santa Cruz		WB/IP/ChIP	Rabbit	pAb
RNA polymerase II(Ser5P)	3E8	04-1572	Millipore	1ug/uL	WB/ChIP	Rat	pAb
RNA polymerase II(Ser2P)	3E10	04-1571	Millipore	1ug/uL	WB	Rat	pAb
S9.6			In-house purified	0.84ug/ml	IF (1:100) IP	Mouse	mAb
SETX	QQ7	SC-100319	Santa Cruz		WB/IP/ChIP	Mouse	mAb
SETX		NB100-57542	Novus	1 µg/µl	WB (1:4000)	Rabbit	mAb

Supplemental Table S2. Related to Figures 1-6. Primers used in this study.

	Forward	Reverse
ACTB_PPP	GAGGGGAGAGGGGGTAAA	AGCCATAAAAGGCAACTTTCG
ACTB_In1	CGGGGTCTTTGTCTGAGC	CAGTTAGCGCCCAAAGGAC
ACTB_In5	GGAGCTGTCACATCCAGGGTC	TGCTGATCCACATCTGCTGG
ACTB_pA	TTACCCAGAGTGCAGGTGTG	CCCCAATAAGCAGGAACAGA
ACTB_TES	GGGACTATTTGGGGGTGTCT	TCCCATAGGTGAAGGCAAAG
GAPDH_PPP	CTCCTGTTCGACAGTCAGC	TTCAGGCCGTCCCTAGC
GAPDH_In5	ATAGGCGAGATCCCTCCAA	TGAAGACGCCAGTGGAC
GAPDH_TES	CCCTGTGCTCAACCAGT	CTCACCTTGACACAAGCC
TEFM_PPP	CTTGGAGATGAGCGGGTCTG	GACAGACGGGAAATCACCCC
TEFM_In2	TGGCCAATGTGGTGAAAGCC	GGGACTACAGGCCCACGCC
TEFM_TES	ACCACATAGACTTTATGACAGAGAA	TCAATCCATGCTTGTGAAGCAAA
CALM3_PPP	TGCGGGCAGTGAGTGTGGAGG	ACGGGGATCAAGGTTCCTCCGG
CALM3_In1	GTTCGGGCCCCTATTGCGCAC	AAAAGCTGGCTCATTCGAGGCACC
CALM3_TES	GCGATGCCCGTTCTCTTGATC	CGCAGGGGAGTGTTGAAGAGAGA
MRPL21_PPP	CGTTACGCACGCGGTTC	GACCGTCAGGGAAGATGCTG
MRPL21_In4	CGGCATTGAGAATGGTTGCC	GCAATGATTGAGGCTCTCCT
MRPL21_TES	ACCAGGTTTCTGTGTTCTGGT	GGCCTGGTGCTTACAGACAT
RPL13A_PPP	ATGGCGGAGGTGCAGGTATG	AGAGAGGGTGCGACCCCATT
RPL13A_In6	AGATTTCAGGCCTGCTGAGG	CCGCAGACCATCGTGAGATA
RPL13A_TES	TGGCGTCTTTGCACTGTGTC	CTACCCTCTTCAAGCTCCTCAC
VCL_PPP	GTGAGGCTGGTTACGCCGAG	CGGGAACCGGCGAAGAGA
VCL_In6	CTCTGGTATCTGAATCTGCTTTCT	AGCAGTATTTGCAATGTTTGGTTT
VCL_TES	AGAACTTTTAGGTCAGGTTTCTCCT	GTTTCCAGATCTTGAGGATTAGTTT

Supplemental Table S3. Related to Figures 1-3, 5-6, S2-S4. Statistical analysis.

**Figure 1**			P value	summary							
**A**	t-test	two-tailed	0.0061	**							
**B**	t-test	two-tailed	0.0087	**							
**C left**						**C right**					
EUFA423 B2 vs EUFA423	Row Factor	Difference	t	P value	Summary	siCtrl vs siBRCA2	Row Factor	Difference	t	P value	Summary
2-way ANOVA	PPP	0.62	3.918	P<0.01	**	2-way ANOVA	PPP	11.67	3.198	P < 0.05	*
2-way ANOVA	In1	0.18	1.137	P > 0.05	ns	2-way ANOVA	In1	-1	0.2741	P > 0.05	ns
2-way ANOVA	pA	-0.125	0.645	P > 0.05	ns	2-way ANOVA	pA	1.667	0.4569	P > 0.05	ns
2-way ANOVA	TES	0.06	0.3792	P > 0.05	ns		TES	2.667	0.7311	P > 0.05	ns
**D left**						**D right**					
EUFA423 B2 vs EUFA423	Row Factor	Difference	t	P value	Summary	siCtrl vs siBRCA2	Row Factor	Difference	t	P value	Summary
2-way ANOVA	PPP	2.3	2.802	P < 0.05	*	2-way ANOVA	PPP	5.767	5.449	P<0.001	***
2-way ANOVA	In5	0.4833	0.5889	P > 0.05	ns	2-way ANOVA	In5	0.03333	0.0315	P > 0.05	ns
2-way ANOVA	TES	0.33	0.4021	P > 0.05	ns	2-way ANOVA	TES	0.6	0.5669	P > 0.05	ns
**E left**						**E right**					
EUFA423 B2 vs EUFA423	Row Factor	Difference	t	P value	Summary	siCtrl vs siBRCA2	Row Factor	Difference	t	P value	Summary
2-way ANOVA	PPP	1.06	0.8644	P > 0.05	*	2-way ANOVA	PPP	30.71	3.878	P<0.01	**
2-way ANOVA	In2	0.16	0.1305	P > 0.05	ns	2-way ANOVA	In2	9.39	1.186	P > 0.05	ns
2-way ANOVA	TES	3.333	2.718	P > 0.05	ns	2-way ANOVA	TES	2.733	0.3451	P > 0.05	ns

**Figure 2**											
**A**						**B**					
EUFA423 B2 vs EUFA423	Row Factor	Difference	t	P value	Summary	EUFA423 B2 vs EUFA423					
2-way ANOVA	PPP	0.835	4.796	P<0.01	**	2-way ANOVA	Row Factor	Difference	t	P value	Summary
2-way ANOVA	In4	-0.05	0.2872	P > 0.05	ns	2-way ANOVA	PPP	7.668	3.998	P<0.01	**
2-way ANOVA	TES	-0.375	2.154	P > 0.05	ns	2-way ANOVA	In2	-0.335	0.1747	P > 0.05	ns
**C**						**D**					
EUFA423 B2 vs EUFA423	Row Factor	Difference	t	P value	Summary	EUFA423 B2 vs EUFA423	Row Factor	Difference	t	P value	Summary
2-way ANOVA	PPP	0.51	3.964	P<0.01	**	2-way ANOVA	PPP	4.765	5.337	P<0.01	**
2-way ANOVA	In6	-0.05667	0.4404	P > 0.05	ns	2-way ANOVA	In6	1.81	2.027	P > 0.05	ns
2-way ANOVA	TES	0.09667	0.7513	P > 0.05	ns	2-way ANOVA	TES	0.39	0.4368	P > 0.05	ns
**E**						**E**					
EUFA423 B2 vs EUFA423	Row Factor	Difference	t	P value	Summary	EUFA423 vs EUFA423/ERRC4	Row Factor	Difference	t	P value	Summary
2-way ANOVA	PPP	1.368	4.925	P<0.01	**	2-way ANOVA	PPP	-2.123	7.335	P<0.001	***
2-way ANOVA	In5	0.7926	2.853	P < 0.05	*	2-way ANOVA	In5	-1.493	5.158	P<0.001	***
2-way ANOVA	TES	0.7592	2.733	P > 0.05	ns	2-way ANOVA	TES	-1.228	4.241	P<0.01	**

**Figure 3**											
**A left**						**A right**					
EUFA423 B2 vs EUFA423	Row Factor	difference	t	P value	summary	EUFA423 B2 vs EUFA423	Row Factor	Difference	t	P value	Summary
2-way ANOVA	PPP	184.3	3.396	P < 0.05	*	2-way ANOVA	PPP	37.67	3.067	P < 0.05	*
2-way ANOVA	In5	2	0.0369	P > 0.05	ns	2-way ANOVA	In5	2.5	0.1662	P > 0.05	ns
2-way ANOVA	TES	62.33	1.148	P > 0.05	ns	2-way ANOVA	TES	0.3333	0.0272	P > 0.05	ns
**B left**						**B right**					
EUFA423 B2 vs EUFA423	Row Factor	Difference	t	P value	Summary	EUFA423 B2 vs EUFA423	Row Factor	Difference	t	P value	Summary
2-way ANOVA	PPP	-0.1767	1.791	P > 0.05	ns	2-way ANOVA	PPP	-0.028	0.4378	P > 0.05	ns
2-way ANOVA	In5	-0.1333	1.352	P > 0.05	ns	2-way ANOVA	In5	-0.099	1.548	P > 0.05	ns
2-way ANOVA	TES	0.04333	0.4394	P > 0.05	ns	2-way ANOVA	TES	-0.09	1.407	P > 0.05	ns

**Figure 5**											
**A left**						**A right**					
siCtrl vs siBRCA2	Row Factor	difference	t	p-value	summary	siCtrl vs siBRCA2	Row Factor	difference	t	p-value	summary
2-way ANOVA	PPP	-31.06	4.395	P<0.01	**	2-way ANOVA	PPP	-3.75	1.594	P > 0.05	ns
2-way ANOVA	In5	-11.46	1.622	P > 0.05	ns	2-way ANOVA	In5	-14.5	6.163	P<0.01	**
2-way ANOVA	TES	-14.94	2.114	P > 0.05	ns	2-way ANOVA	TES	-10.15	4.314	P < 0.05	*
**B left**						**B right**					
EUFA423 B2 vs EUFA423	Row Factor	difference	t	p-value	summary	EUFA423 B2 vs EUFA423	Row Factor	difference	t	p-value	summary
2-way ANOVA	PPP	11.67	3.198	P < 0.05	*	2-way ANOVA	PPP	-26.37	4.626	P<0.01	**
2-way ANOVA	In5	-14.55	2.143	P > 0.05	ns	2-way ANOVA	In5	-27.3	4.79	P<0.01	**
2-way ANOVA	TES	-13.29	1.958	P > 0.05	ns	2-way ANOVA	TES	-40.43	7.095	P<0.001	***
**C**						**D**					
siCtrl vs siBRCA2	Row Factor	difference	t	p-value	summary	EUFA423 B2 vs EUFA423	Row Factor	difference	t	p-value	summary
2-way ANOVA	PPP	0	0	P > 0.05	ns	2-way ANOVA	PPP	0	0	P > 0.05	ns
2-way ANOVA	In5	-0.5025	6.185	P<0.001	***	2-way ANOVA	In5	-0.7236	3.049	P < 0.05	*
2-way ANOVA	TES	-0.4417	5.436	P<0.001	***	2-way ANOVA	TES	-0.7155	3.015	P < 0.05	*

**Figure 6**					
**A**					
siCtrl vs siBRCA2	Row Factor	Difference	t	P value	Summary
2-way ANOVA	PPP	6.888	1.707	P < 0.05	*
2-way ANOVA	In5	1.533	0.4103	P > 0.05	ns
2-way ANOVA	TES	1.095	0.2931	P > 0.05	ns
siCtrl vs siPAF1	Row Factor	Difference	t	P value	Summary
2-way ANOVA	PPP	12.06	3.229	P<0.01	**
2-way ANOVA	In5	5.233	1.401	P > 0.05	ns
2-way ANOVA	TES	4.67	1.25	P > 0.05	ns
siCtrl vs siBRCA2/siPAF1	Row Factor	Difference	t	P value	Summary
2-way ANOVA	PPP	11.09	2.748	P < 0.05	*
2-way ANOVA	In5	2.333	0.5781	P > 0.05	ns
2-way ANOVA	TES	1.812	0.449	P > 0.05	ns
**C**					
EUFA423 B2 vs EUFA423	Row Factor	Difference	t	P value	Summary
2-way ANOVA	PPP	3.015	3.78	P < 0.05	*
2-way ANOVA	In5	-0.15	0.1881	P > 0.05	ns
2-way ANOVA	TES	-0.4	0.5015	P > 0.05	ns
EUFA423 B2 vs EUFA423 + PAF1	Row Factor	Difference	t	P value	Summary
2-way ANOVA	PPP	-0.09	0.1128	P > 0.05	ns
2-way ANOVA	In5	-0.885	1.11	P > 0.05	ns
2-way ANOVA	TES	-1.38	1.73	P > 0.05	ns

Supplemental Figure 2

A

siCtrl vs siBRCA1	Row Factor	Difference	t	P value	Summary
2-way ANOVA	PPP	-0.1537	1.474	P > 0.05	ns
2-way ANOVA	In 1	0	0	P > 0.05	ns
2-way ANOVA	pA	0.09445	0.9058	P > 0.05	ns
2-way ANOVA	TES	0.319	3.06	P < 0.05	*

B

siCtrl vs siBRCA2	Row Factor	Difference	t	P value	Summary
2-way ANOVA	PPP	4.615	4.999	P<0.01	**
2-way ANOVA	In5	1.8	1.95	P > 0.05	ns
2-way ANOVA	TES	3.1	3.358	P < 0.05	***

C

siCtrl vs siBRCA2	Row Factor	Difference	t	P value	Summary
2-way ANOVA	PPP	7.667	4.193	P<0.01	**
2-way ANOVA	In5	3.667	2.006	P > 0.05	ns
2-way ANOVA	TES	14.33	7.84	P < 0.05	*

Supplemental Figure 3

A

EUFA423 B2 vs EUFA423	Row Factor	Difference	t	P value	Summary
2-way ANOVA	PPP	-15.64	2.84	P < 0.05	*
2-way ANOVA	In2	-17.09	2.776	P > 0.05	ns
2-way ANOVA	TES	-1.16	0.2107	P > 0.05	ns

B

EUFA423 B2 vs EUFA423	Row Factor	Difference	t	P value	Summary
2-way ANOVA	PPP	-16.81	10.4	P<0.001	***
2-way ANOVA	In2	0.8933	0.5528	P > 0.05	ns
2-way ANOVA	TES	-0.1267	0.0784	P > 0.05	ns

C

EUFA423 B2 vs EUFA423	Row Factor	Difference	t	P value	Summary
2-way ANOVA	PPP	-3.123	3.874	P<0.01	**
2-way ANOVA	In6	0.05333	0.0662	P > 0.05	ns
2-way ANOVA	TES	0.15	0.186	P > 0.05	ns

D

EUFA423 B2 vs EUFA423	Row Factor	Difference	t	P value	Summary
2-way ANOVA	PPP	-9.317	2.844	P < 0.05	*
2-way ANOVA	In6	-0.81	0.2473	P > 0.05	ns
2-way ANOVA	TES	-1.113	0.3399	P > 0.05	ns

Supplemental Figure 4

Unpaired t test	
P value	0.0035
P value summary	**
